# Systems genomics approaches provide new insights into *Arabidopsis thaliana* root growth regulation under combinatorial mineral nutrient limitation

**DOI:** 10.1371/journal.pgen.1008392

**Published:** 2019-11-06

**Authors:** Nadia Bouain, Arthur Korte, Santosh B. Satbhai, Hye-In Nam, Seung Y. Rhee, Wolfgang Busch, Hatem Rouached

**Affiliations:** 1 BPMP, Univ Montpellier, CNRS, INRA, SupAgro, Montpellier, France; 2 Evolutionary Genomics, Center for Computational and Theoretical Biology (CCTB), University Würzburg, Würzburg, Germany; 3 Gregor Mendel Institute (GMI), Austrian Academy of Sciences, Vienna Biocenter (VBC), Vienna, Austria; 4 Plant Molecular and Cellular Biology Laboratory, and Integrative Biology Laboratory, Salk Institute for Biological Studies, La Jolla, California, United States of America; 5 Department of Plant Biology, Carnegie Institution for Science, Stanford, California, United States of America; The University of North Carolina at Chapel Hill, UNITED STATES

## Abstract

The molecular mechanisms by which plants modulate their root growth rate (RGR) in response to nutrient deficiency are largely unknown. Using *Arabidopsis thaliana* accessions, we analyzed RGR variation under combinatorial mineral nutrient deficiencies involving phosphorus (P), iron (Fe), and zinc (Zn). While -P stimulated early RGR of most accessions, -Fe or -Zn reduced it. The combination of either -P-Fe or -P-Zn led to suppression of the growth inhibition exerted by -Fe or -Zn alone. Surprisingly, root growth responses of the reference accession Columbia (Col-0) were not representative of the species under -P nor -Zn. Using a systems approach that combines GWAS, network-based candidate identification, and reverse genetic screen, we identified new genes that regulate root growth in -P-Fe: *VIM1*, *FH6*, and *VDAC3*. Our findings provide a framework to systematically identifying favorable allelic variations to improve root growth, and to better understand how plants sense and respond to multiple environmental cues.

## Author summary

Plants thrive in highly heterogenous soils. How they compute a multitude of contrasting stimuli and mount an adaptive response without a centralized information processing unit is an intriguing question. For instance, below ground, roots can sense and respond to the single or multiple nutrient stresses, and adjust its growth rate accordingly. Nevertheless, the genetic architecture of root growth responses under single and combined stress remains poorly understood. To fill this gap in our understanding about such crucial phenomenon for plant survival, we explored the natural variation of root growth rate (RGR) in Arabidopsis grown under single and combined nutritional stress, including deficiencies of iron (-Fe), zinc (-Zn), phosphate and iron (-P-Fe) and phosphate and zinc (-P-Zn). Our GWAS revealed distinct genetic architectures underlying root growth responses to single or combined nutrient stresses. By integrating GWAS and coexpression networks, we identified and validated genes controlling the variation of root growth to combined nutrient-deficiency, namely *VARIANT IN METHYLATION 1*, *FORMIN-LIKE-PROTEIN-6* and *VOLTAGE-DEPENDENT ANION-SELECTIVE CHANNEL PROTEIN 3*. Our findings provide a framework to accelerate future research aiming at better understanding how plants sense and respond to multiple environmental inputs, and promise to help designing new agronomical and biotechnological strategies to improve root growth.

## Introduction

Global climate change and population increase pose a tremendous challenge and prompt an urgent need for efficient agriculture and food production. The world population is projected to exceed 9.8 billion by 2050, and global food production will have to increase by 70% to sustain this population [1]. At the same time, climate change is associated with a decrease of important micronutrients such as iron (Fe) and zinc (Zn) in staple foods such as rice [2]. The bio-availability of Fe and Zn is often limited in the soil, leading to reductions in growth, crop yield, and quality. As plants are the entry point of these elements in the food web, low accumulation of these elements in plants is associated with malnutrition in humans. Zn and Fe deficiencies are estimated to affect up to 2 billion people worldwide [3]. Moreover, modern industrialized agriculture has created a strong demand and dependency for fertilizers, posing the danger of acute limitation of non-renewable fertilizer components, in particular phosphorus (P) [4,5]. All these nutrients (P, Zn and Fe) are taken-up by plants at the root-soil interface, and plants often face conditions in which one or more of these elements are limiting [6,7]. Thus, improving the capacity of plants to absorb these nutrients from the soil is a major goal of crop improvement. The development of crops with higher tolerance for individual and multiple nutrient stresses is a direction towards more sustainable and efficient agriculture.

P is an essential macronutrient for plant growth and development. P is a critical component of many macromolecules (e.g. DNA), energy sources (e.g. ATP) and regulation of signal transduction via phosphorylation. Plants acquire P as soluble inorganic phosphate (Pi) by a suite of high affinity phosphate transporters in the root [8]. In soil, P distribution is heterogeneous [9], and is usually found in shallow soil layers [10]. To cope with P heterogeneity in soil, plants increase root growth in shallow soil layers that promote topsoil foraging, thereby conferring an advantage for P acquisition [11,12].

The effects of P deficiency (-P) on the root system have been studied in many plant species. For the model plant *Arabidopsis thaliana* (*A*. *thaliana*), the accession Columbia (Col-0) is commonly considered as the reference [13]. Changes that occur in root architecture in -P consist of reduction of primary root growth (PRG), an increase in the growth of secondary roots, and an increase in root hair length and density (for review, see [14]). A handful of genes involved in PRG under -P have been cloned. Under -P, mutants of these genes are characterized either by their ability to maintain PRG such as the *low phosphate root 1* (*lpr1*) mutants [15], or by a strong inhibitory effect of the PRG (hypersensitivity) such as the *phosphate deficiency response 2* (*pdr2*) mutants [16] and *hypersensitive to pi starvation 7* (*hps7*) [17]. More genes involved in inhibition of PRG were recently identified, including a transcription factor SENSITIVE TO PROTON RHIZOTOXICITY (STOP1) and its target ALUMINUM ACTIVATED MALATE TRANSPORTER 1 (ALMT1) [18,19]. In addition to genetic screens for mutants affected in their response to -P, *A*. *thaliana* has natural accessions that are either oversensitive (e.g. Shahdara) or more tolerant (e.g. Landsberg *erecta*) to -P compared to Col-0 [20]. The existence of natural variation in primary root growth in response to -P indicates the potential for discovering new genes that control this trait via quantitative genetic approaches.

Fe and Zn are involved in vital biological processes ensuring proper functioning of the cell. Fe is a cofactor for numerous enzymes and part of Fe–S clusters that are a major sink for Fe and essential for many important cellular processes such as photosynthesis and respiration [21,22]. Similarly, Zn functions as a cofactor for hundreds of enzymes [23]. Since Zn and Fe are taken up by roots, improvement of root growth could help increase Zn and Fe content in plants. In Col-0, deficiencies in Fe and Zn impose a change in root architecture in a contrasting manner [6]. While Fe deficiency (-Fe) inhibits root elongation, Zn deficiency (-Zn) slightly promotes early primary root growth [6]. Only few genes that regulate primary root growth under -Fe or -Zn conditions have been identified. For instance, mutations in the BASIC HELIX-LOOP-HELIX-TYPE transcription factors POPEYE [24], or the two interacting transcription factors, bHLH34 and bHLH104 [25], inhibited the primary root growth in -Fe. Moreover, very few genetic variants have been identified that contribute to contrasting growth responses to -Fe and -Zn that can be observed for natural Arabidopsis accessions [26,27]. More genes and genetic variants that control root architecture under -Fe and -Zn conditions remain to be identified. The convenience of genome-wide association studies (GWAS) in *A*. *thaliana* offers an opportunity to explore the genetic diversity in mineral nutrient responses and interactions between nutrients in natural populations, in order to identify genes and alleles controlling these traits.

Recent research has shown that nutrient homeostasis by interaction between nutrients is a general rule in plants rather than an exception (for review, [28,29]). P and Zn or P and Fe interact in the plant, and these interactions are visible at the molecular level [6], where the deficiency of one element induces or represses the expression of genes involved in the regulation of the other element [29–33]. Downstream responses to -P or -Fe may share hormone signals, such as cytokinin. For example, cytokinin signaling, mediated by the cytokinin receptors CYTOKININ RESPONSE 1/WOODEN LEG/ARABIDOPSIS HISTIDINE KINASE 4 (CRE1/WOL/AHK4), is involved in the response to -P or -Fe in *A*. *thaliana* (Col-0) [34–36]. The outcome of the interaction of these nutrients is also visible at the morphological level [32]. Perhaps the most prominent example is the -P-Fe interaction and its effect on primary root growth. Primary root growth inhibition in response to -P has been proposed to be the result of Fe “toxicity” [37] and it has been suggested that the inhibition of Arabidopsis (Col-0) primary root growth under -P is due to a presumed overabundance of available Fe, and is not solely due to -P alone [37]. This Fe overabundance was proposed to depend on malate exudation and presumably malate chelating Fe. The malate transporter ALMT1 was shown to be involved in this process, likely by promoting Fe accumulation in the root meristem causing the inhibition of cell expansion under -P [18,19]. Fe accumulation in the root tip of P-deficient plants was proposed to be the cause for the differentiation of the apical root meristem—possibly through a prevention of the symplastic cell-to-cell communication as a consequence of callose deposition [18]. The PDR2–LPR1 module was proposed to mediate this callose accumulation in root meristems experiencing -P [19]. It has been proposed that the CLAVATA3/ESR (CLE)-related protein 14 precursor (CLE14) is the signal that triggers full root meristem differentiation in -P through CLV2/PEPR2 receptors [38]. It has been shown that the CLE14 pathway acts downstream of LPR1/LPR2 [38]. In contrast, these cellular hallmarks of -P were not observed under simultaneous P and Fe deficiency. The callose deposition was not detected and primary root growth was comparable to plants grown under complete medium. Thus far, these two modules LPR1-PDR2 and STOP1-ALMT1 have been used to explain how P and Fe signals shape primary root growth under P and Fe deficiencies [17,18].

Plants have evolved mechanisms to sense and respond to nutrient deficiency early in their life cycle. Soon after germination, roots are the main targets of nutrient deficiency stress. Root growth responses to nutrient changes are genetically determined, and vary between and within plant species [39]. Identifying genes and mechanisms that underlie natural variation of root growth has become possible through large-scale phenotyping and various mapping approaches [40].

Here we set out to investigate variation in the primary root growth rate (RGR) of a panel of natural accessions in *A*. *thaliana* under single and combined deficiencies of P, Fe, and Zn. We used GWAS to identify candidate genes involved in RGR regulation under each of the growth conditions tested. Finally, we used a network biology driven search using these candidate genes to identify potential networks and processes that are relevant for determining RGR under the nutrient deficiency conditions. Using mutant analysis, we confirmed three new genes involved in regulating PRG under the combined nutrient stress -P-Fe. These genes are *VARIANT IN METHYLATION 1 (VIM1)*, *FORMIN-LIKE PROTEIN 6 (FH6) and VOLTAGE-DEPENDENT ANION-SELECTIVE CHANNEL PROTEIN 3 (VDAC3)*. Taken together, our findings shed light on the regulation of root growth by combinatorial mineral nutrient cues and provide a foundation for guiding new agronomical and biotechnological strategies to improve root growth.

## Results

### RGR responds distinctly to single and combined nutrient deficiencies in a genotype dependent manner

**Fig 1:**
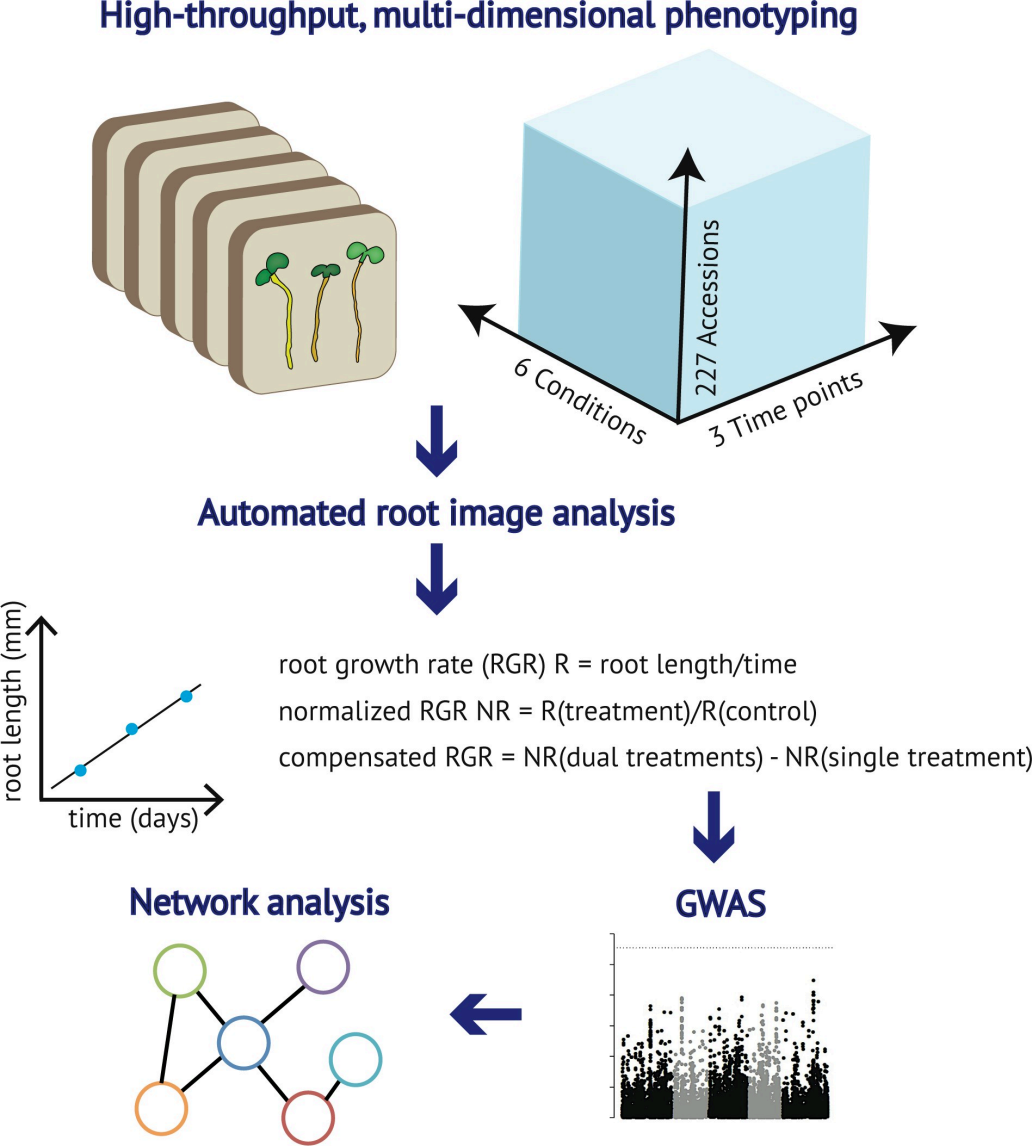
A systems framework to study root growth under mineral limitation. (A) Design of the experimental set up. 227 *Arabidopsis thaliana* accessions were grown on vertical agar plates containing different media, including control (Ct), deficiency of phosphorus (-P), iron (-Fe), zinc (-Zn), phosphorus and iron (-P-Fe), or phosphorus and zinc (-P-Zn). Seedlings were daily imaged and primary root length (PRL) of 3-, 4-, 5-day-old seedlings was determined. (B) The root growth rate (RGR) was determined by conducting linear regression on PRL of twelve replicates per accession and for each treatment. Five GWAS were performed using on normalized RGRs, which were obtained for each accession by calculating the ratio between RGR on each nutrient deficiency condition divided by the value on the control condition (Ct). GWAS was also performed on the variation of normalized RGR on -P-Fe and -Fe, or -P-Zn and–Zn by the value on control condition (Ct) and expressed as ΔRGR_(-P-Fe, -Fe)_ = RGR_(-P-Fe/Ct)_—RGR_(-Fe/Ct)_, and for -P-Zn and–Zn ΔRGR_(-P-Zn, -Zn)_ = RGR_(-P-Zn/Ct)_—RGR_(-Zn/Ct)_. (C) The significance of associations between phenotypes and the single nucleotide polymorphisms (SNPs) markers was evaluated using linear mixed model. The GWAS candidate genes for a given RGR trait were used to identify a gene connected with one another, which form a molecular pathway, using a machine learning algorithms to infer co-functional links from genomics data, AraNetv2 [54].

Natural variation of root system architecture has been reported for -P [20,41,42] as well as for -Fe [26] and -Zn [27], yet never for their combination (-P-Fe or -P-Zn). In particular, root growth rate (RGR) as a trait has not been evaluated under any nutritional stresses in a population of natural accessions. Therefore, we set out to explore the natural variation of RGR in *A*. *thaliana* by investigating the RGR of 227 genetically diverse natural accessions from the RegMap population [43] (S1 Table) grown on different media. We tested six growth conditions: control (Ct), deficiency of P (-P), Fe (-Fe), Zn (-Zn), P and Fe (-P-Fe), and P and Zn (-P-Zn) (Fig 1). Seedlings were imaged daily at the same time, and the primary root length (PRL) was determined using the BRAT software [40]. For each accession, we recorded the primary root length (PRL) of three, four and five-day old seedlings (S1 Table). We first examined the PRL of Columbia-0 (Col-0) accession under all growth conditions tested, as this accession is most widely used in Arabidopsis research. Consistent with previous studies [37,44], -P or -Fe caused a reduction of the Col-0 PRL, and the reduced PRL observed in -P was not observed in -P-Fe (S1 Fig). A similar suppression of -P dependent growth rate reduction was observed in -P-Zn (S1 Fig). Overall, the response of PRL in Col-0 was consistent with previous reports, thus validating our experimental setup.

**Fig 2:**
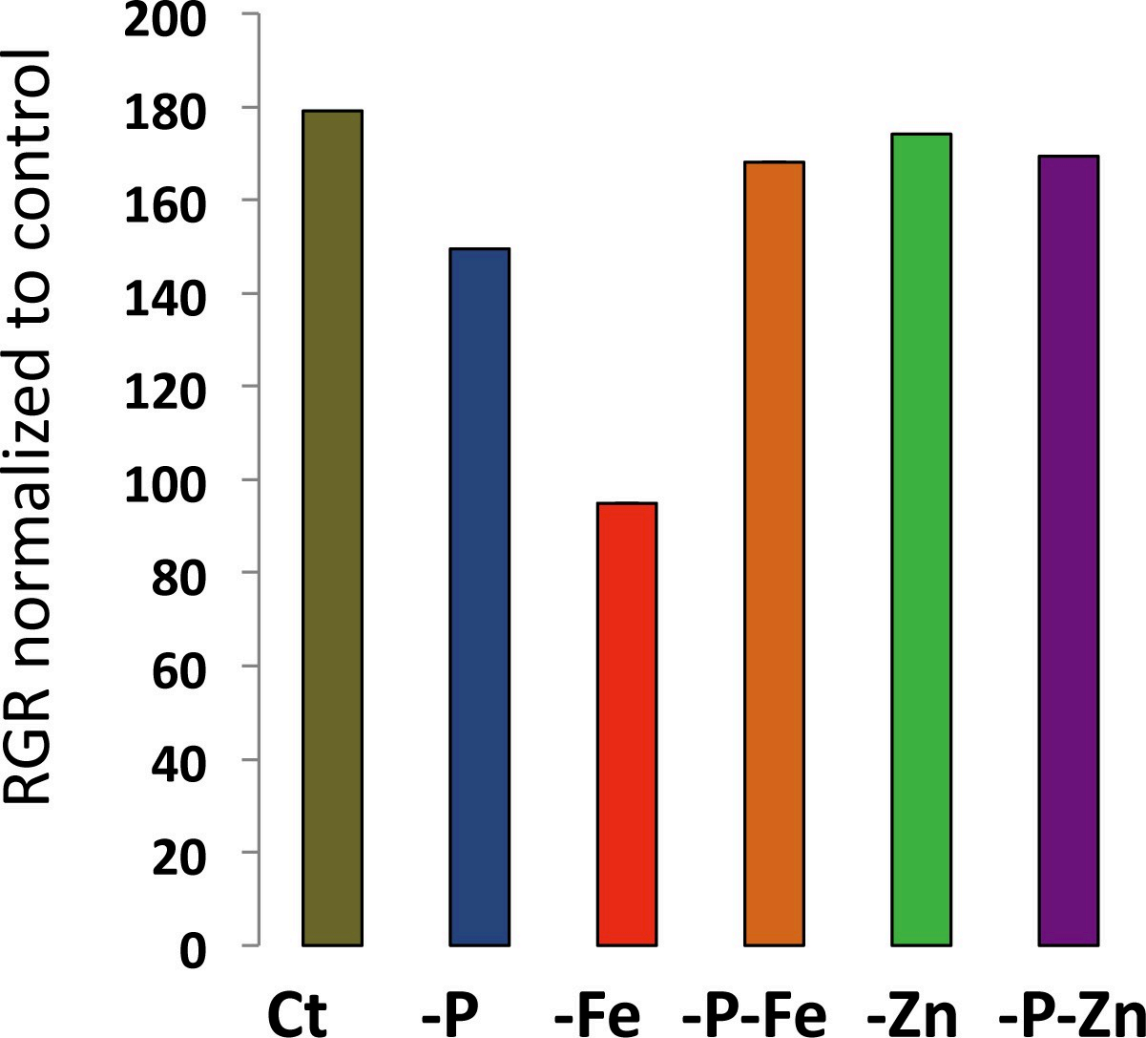
Effect of single and double deficiencies of iron, zinc and phosphorus on the root growth rate of the *Arabidopsis thaliana* reference accession Col-0. Seeds of the *A*. *thaliana* Col-0 accession were germinated on six different nutrient conditions: control (Ct), deficiency of P (-P), Fe (-Fe), Zn (-Zn), P and Fe (-P-Fe), and P and Zn (-P-Zn). The primary root length of 3-, 4-, and 5-day-old seedlings on each treatment was used to determine the regression coefficient.

**Fig 3:**
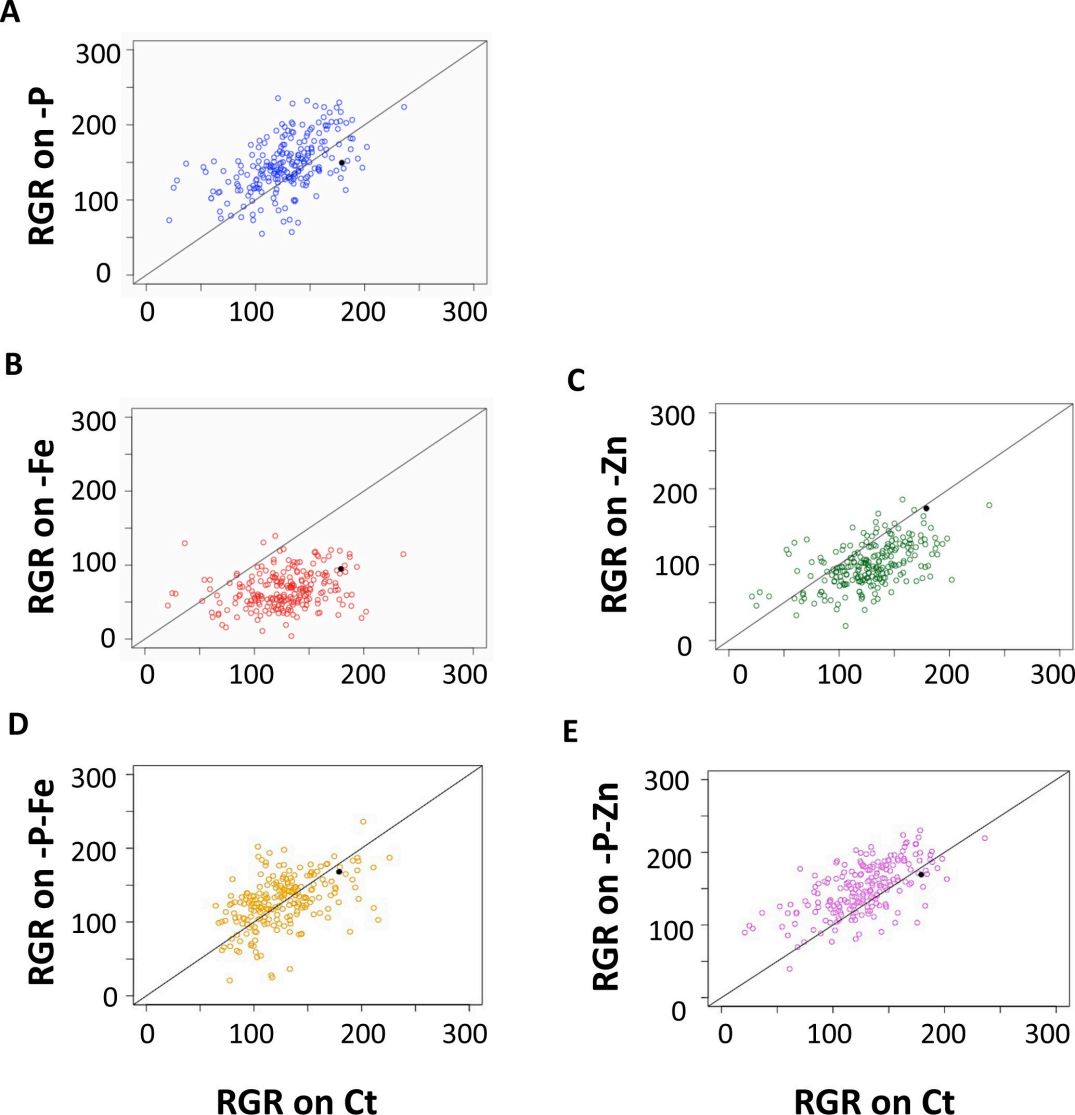
Natural variation of 227 *Arabidopsis thaliana* accessions in their responses to single and double deficiencies in iron, zinc and phosphorus. (A) Relationship between root growth rate (RGR) under phosphate deficiency (-P) and RGR under control condition (Ct). (B) Relationship between RGR under iron deficiency (-Fe) and root growth in Ct. (C) Relationship between RGR under zinc deficiency (-Zn) and RGR in Ct. (D) Relationship between RGR in -P-Fe and RGR in Ct. (E) Relationship between root growth rate under in -P-Zn and RGR in Ct. Each data point represents the RGR estimated from a pool of plants (median n in each treatment > 10). The solid black dot represents the Col-0 reference accession. The solid black line represents the diagonal (i.e. the growth that would be expected without any response) and not the results of a regression analysis.

We next examined the whole set of accessions. To account for differences in germination of the accessions, we used RGR for all further comparisons. For this we conducted linear regression on the root length measurements, whereby the regression coefficient provided an estimate of the root growth rate in different treatments (Fig 1, S1 and S2 Tables). Under the assumption of linear growth during this early growth period, the regression coefficient on the replicates is an ideal measurement, and indeed the estimated slopes of the RGR are significant for most accessions in most treatments (99.5% for -P, 98.7% for -P-Zn%, 97,8% for -P-Fe, 96,5% for Ct, 93% for -Fe, 92,5% for -P-Zn) (S1 Table, columns 21–32). Compared with Ct condition the RGR of Col-0 was reduced in -P or -Fe conditions, but not in -Zn, -P-Fe or -P-Zn conditions (Fig 2). Because the data sets were obtained simultaneously, they constitute a unique resource for comparing the RGRs between accessions. Our analysis revealed a large variation of RGR among the accessions in the control condition (Ct) and in response to each of the nutrient deficiency conditions (Fig 3). To test whether the variation of root growth is genetically determined, we analyzed the heritability (H^2^) [45] of RGR using estimates from the mixed model [46]. We found that the phenotypic variation of RGR in response to the different conditions is a heritable trait displaying broad sense heritabilites from 10% (RGR response -Fe) to 80% (RGR on control conditions) (S3 Table). Like Col-0, the RGR of most accessions was reduced by -Fe treatment, and the extreme accessions included Zu-1, MIR-0, Rmx-A02, Edinburgh-5, and Ove-0. However, -Zn reduced RGR in most accessions, and the extreme accessions included Shahdara, Sq-1, Sapporo-0, and Si-0. Here Col-0, which slightly increases RGR, was the exception to the rule. We found a similar surprise for RGR in -P, as Col-0 showed a reduction in their RGR while most accessions behaved in an opposite manner; their RGR was increased compared to Ct. When grown under combined nutrient deficiency (-P-Fe and -P-Zn), most accessions showed an RGR that was similar to the -P effect and distinct from -Fe or -Zn alone (Fig 3). P deficiency alleviated the RGR reduction mediated by Fe or Zn deficiency. Taken together, our analysis revealed that -P generally promoted early primary root growth whereas -Fe or -Zn reduced it in *A*. *thaliana*. In addition, removing P alleviated the root growth reduction brought on by Fe or Zn deficiency. This general response varied across natural accessions in response to single or combined nutrient deficiencies. Importantly, Col-0 was not the best representative accession for the responses in -P or -Zn conditions.

### Distinct genetic architectures underlie the response to single and combined nutrient stresses in *Arabidopsis thaliana*

**Fig 4:**
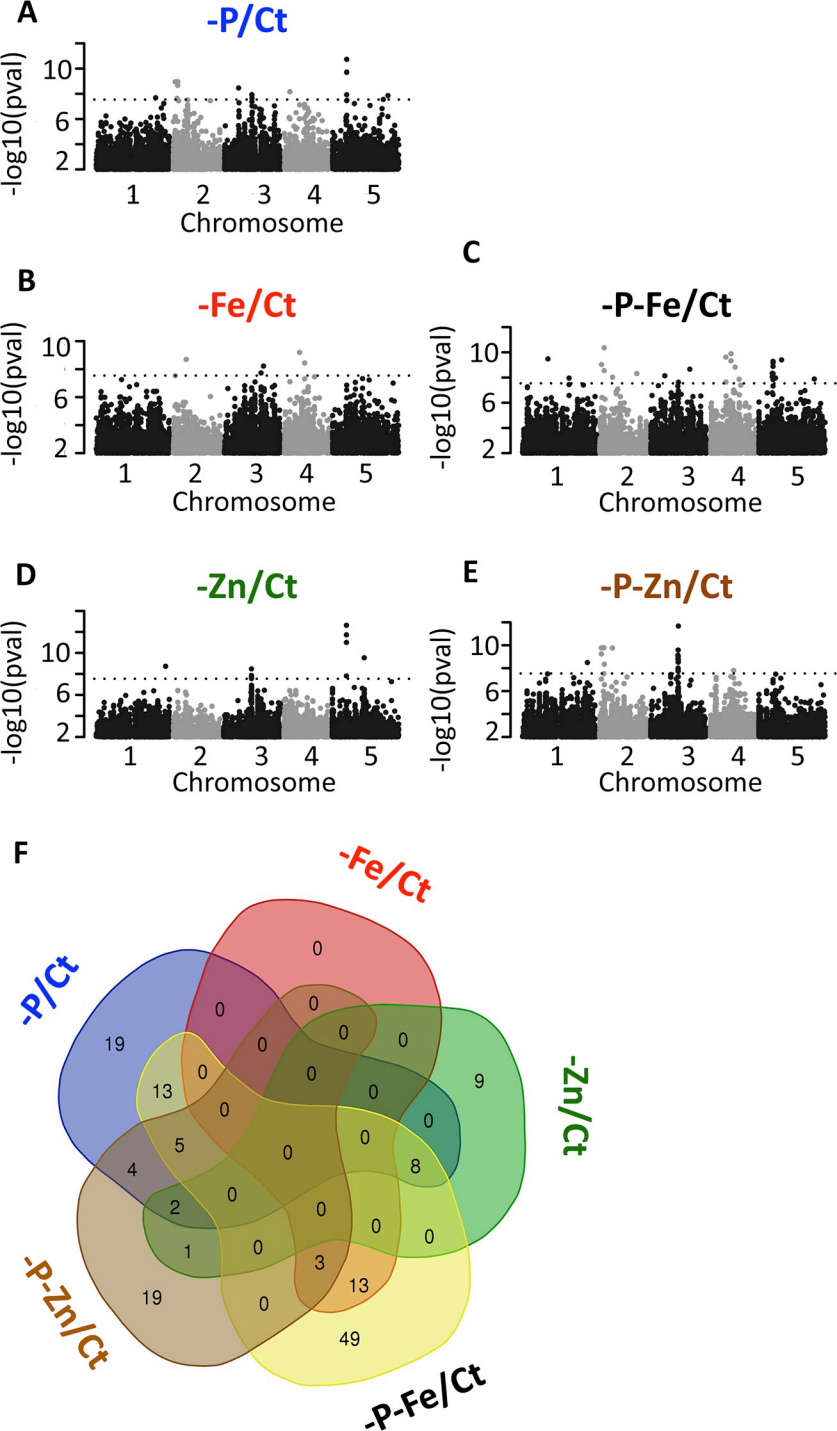
Genetic architectures of root growth rate control in response to single and double deficiencies of iron, zinc and phosphorus from GWAS. (A-E) Genome-wide distribution of the −log10 P-values of SNP/phenotype associations using a linear mixed model method that corrects for population structure (AMM). SNPs associated with the root growth rate under the deficiency of -P (A), -Fe (B) -Zn (C), -P-Fe (D), or -P-Zn (E) compared to control condition (Ct) are presented. SNPs are plotted according to their position along the chromosomes. Plotting colors alternate between black and grey in order to facilitate the visualization of each of the five chromosomes. 5% Bonferroni threshold is indicated by black dashed line. (F) Venn Diagram of GWAS candidate genes underlying Arabidopsis RGR under the different nutrient-deficient conditions: -P, -Fe, -Zn, -P-Fe and -P-Zn conditions. The Venn diagram was generated using a web-based tool for the analysis of sets through Venn diagrams (InteractiVenn) [79].

To determine the genomic loci that control RGR in response to mineral nutrient deficiencies, we performed GWAS on RGR. To assess the genomic architecture that is specific for the respective nutrient deficient condition, we normalized the RGRs prior to the analyses (Fig 4, S2 Table). For each accession, normalized RGR was defined as the ratio between RGR from each nutrient deficiency condition divided by the RGR from the control condition (Ct). The significance of associations between phenotypes and the single nucleotide polymorphism (SNP) markers was evaluated using a linear mixed model (a modified version of EMMA) [46] that corrects for population structure. All considered normalized RGRs show low but significant estimated heritabilities (H^2^) (S3 Table). We next used ~1.7 million markers with a minor allele frequency of at least 5% in the population and corrected the associated p-values for multiple hypothesis testing using a 5% Bonferroni threshold (Fig 4A–4E). Using this conservative threshold, and by taking into account genes present in a 10kb window surrounding the underlying significant markers — acknowledging the rapid decay of linkage disequilibrium (LD) in the Arabidopsis population [47]—we identified a list of 145 candidate genes (S4 Table) corresponding to 87 significant SNPs in 32 distinct genomic regions (S5 Table). Of these, 96 genes (49 SNPs) were associated with only one trait, while 31 genes (19 SNPs) were associated with two traits, and 18 genes (19 SNPs) were associated with 3 traits (S4 Table). Among these candidates, some are known to be involved in regulating root growth under mineral nutrient deficiency. For instance, the *CLV2* gene is known to trigger full root meristem differentiation under -P [38] and was identified in our GWAS RGR on -P (p-value = 2.1x10^-8^). Many candidate genes related to different classes of hormone signaling were also identified, including genes involved in auxin, gibberellin, cytokinin, ABA, and brassinosteroid pathways. For instance, the gene *BRASSINAZOLE-RESISTANT 1* (*BZR1*, p-value = 1.9x10^-9^) was identified to associate with the regulation of RGR under -Zn. Interestingly, there was hardly any overlap among the gene lists of single nutrient deficiencies (-P, -Zn, and -Fe) (Fig 4F). No common gene was detected between -P and -Fe, nor between -Fe and -Zn (Fig 4F, S4 Table). Only in the case of -P and -Zn, two overlapping regions (tagged by 17 SNPs) corresponding to 10 candidate genes were detected. The first region was significantly associated with -P-Zn, and the second region was associated with -P-Fe, in addition to -P and -Zn (S6 Table). The three associated SNPs in the first region, common between -P, -Zn, and-P-Zn, are in complete LD and tag the genes AT3G29570 and AT3G29575 (p-values: -P (2.1x10^-8^), -Zn (3.3x10^-9^), and -P-Zn (9.0x10^-10^)). AT3G29575 is a member of the family of ABI five binding proteins (AFPs) and is involved in the stress response of germinating seeds and seedlings through modulation of ABA signaling [48]. It is worth to mention that the results from GWAS on normalized RGRs showed no correlation with those obtained on non-normalized RGRs (S2 Fig), and no significant associations were found using the non-normalized RGRs. One reason for this might be the complex nature of the non-normalized RGR, where many factors could contribute to the phenotype, while the normalization enabled us to identify genetic factors that are specific for the response to the respective nutrient deficiency. Taken together, our GWAS analysis allowed the identification of interesting candidate genes that may underlie the natural variation of RGR in response to mineral nutrient deficiencies, and revealed that root growth responses to single stresses and to their combinations might be regulated by distinct genetic programs rather than being regulated in a simple additive manner.

### The negative regulation of RGR by iron and zinc deficiencies is suppressed when combined with phosphate deficiency

**Fig 5:**
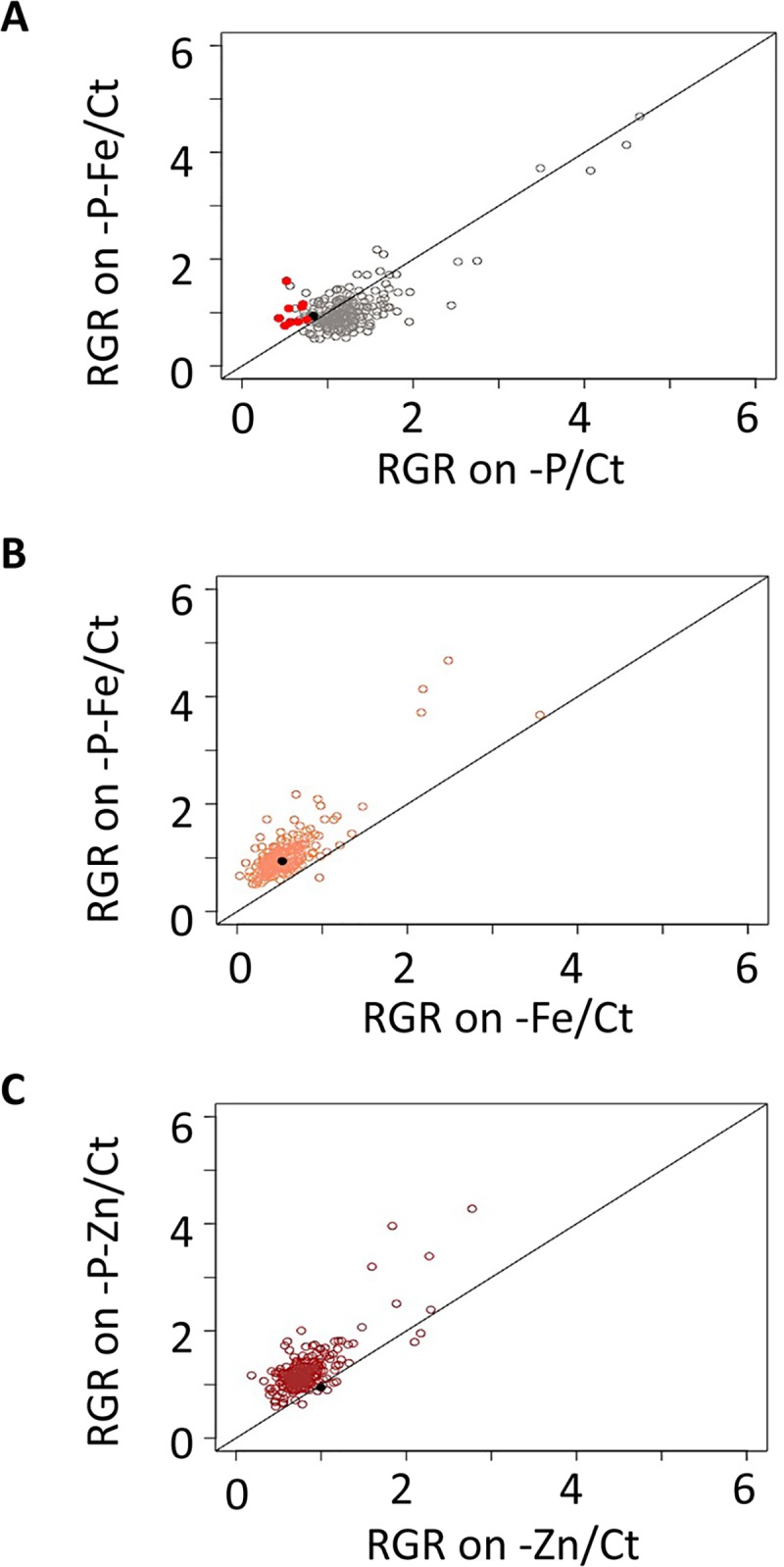
P deficiency suppresses negative effects of Fe and Zn deficiency on the primary root growth rate of most *Arabidopsis thaliana* accessions. The root growth rate (RGR) of 227 accessions of *Arabidopsis thaliana* grown under each of the nutrient-deficient conditions was normalized to the corresponding values under control conditions (Ct). (A) Relationship between RGR under combined phosphorus and iron deficiency (-P-Fe/Ct) and RGR under phosphorus deficiency (-P/Ct). The red dots represent the accessions that showed limited RGR by -P considering one standard deviation away from the mean with 15.8% confidence limit to the RGR data, and which are promoted in -P-Fe. (B) Relationship between RGR under phosphorus and iron deficiency (-P-Fe/Ct) and RGR under iron deficiency (-Fe/Ct). (C) Relationship between root RGR under phosphorus and zinc deficiency (-P-Zn/Ct) and RGR under zinc deficiency (-Zn/Ct). Each data point was obtained from the analysis of RGR from a pool of plants (n ≥ 9). The solid black dot represents the Col-0 reference accession. The solid black lines represent the diagonal.

**Fig 6:**
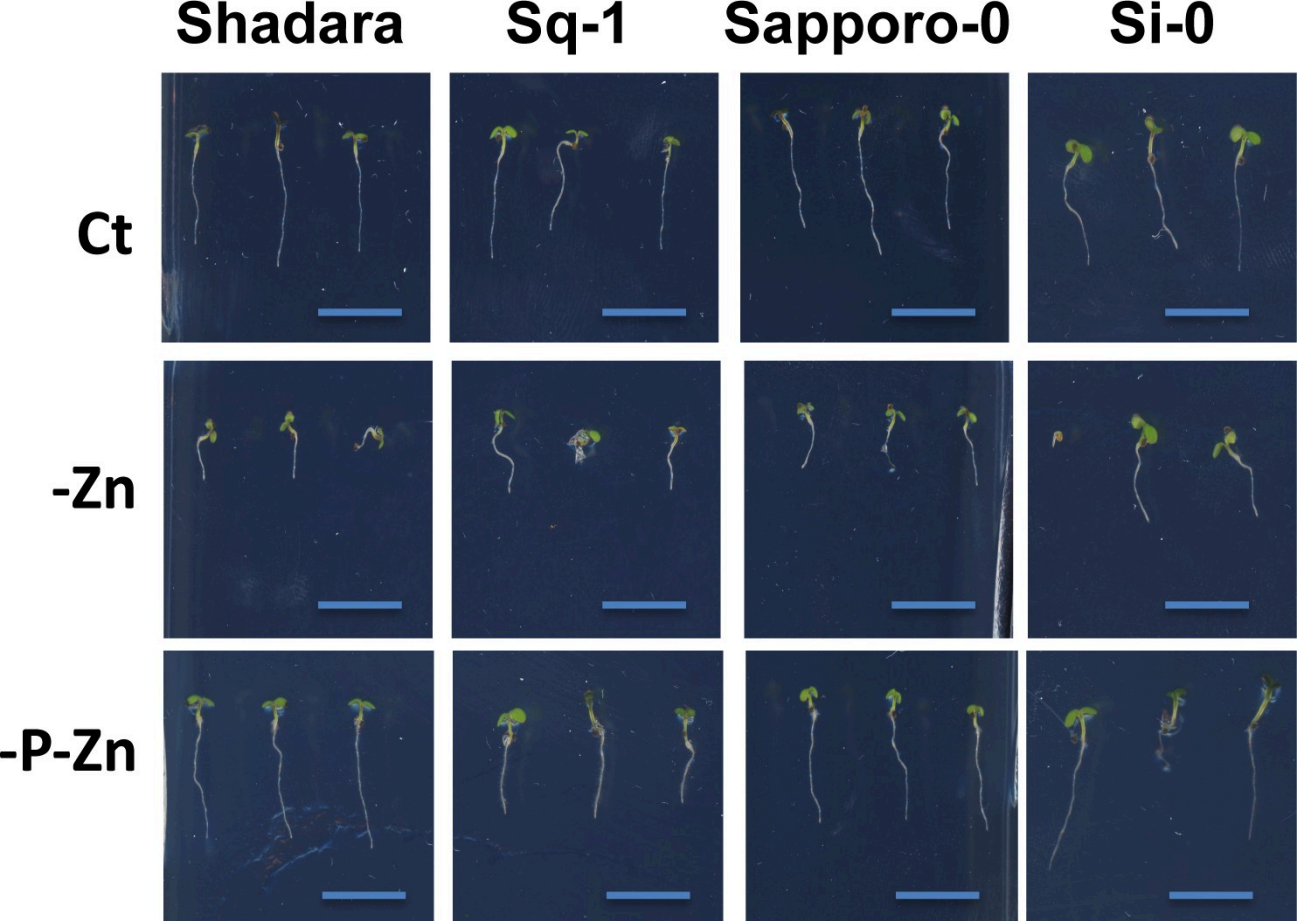
Combined phosphorus and zinc deficiency promotes primary root growth rate of most *Arabidopsis thaliana* accessions. Representative images of contrasting primary root growth phenotype (5 days) of four *Arabidopsis thaliana* accessions (Shahdara, Sq-1, Sapporo-0, Si-0) grown on vertical agar plates in the presence of zinc (+Zn), in the absence of zinc (-Zn), or the absence of both phosphorus and zinc (-P-Zn).

In line with the literature, we found that Col-0 plants grown under -P-Fe have longer primary roots compared to plants grown either on -P or -Fe (Fig 2). But, whether this compensation is a general mechanism of adaptation or just an exception for a few accessions, and whether -P could also alleviate -Zn’s negative effect on RGR in Arabidopsis remained unknown. We therefore set out to answer these questions. We compared normalized RGRs on combined nutrient stress (-P-Fe/Ct or -P-Zn/Ct) to normalized RGRs on single nutrient stress (-P/Ct, -Fe/Ct, or -Zn/Ct) (Fig 5A–5C). First, only a subset of accessions displayed a reduction of RGR on -P (e.g. Col-0, Sorbo, Rd-0, Rmx-A180, Mr-0, Hau-0, Got-22, Mdn-1, RRS-10) and in each of these accessions this reduction was abolished by the combined stress -P-Fe (Fig 5A, black dot for Col-0 and red dots for the rest). Second, the reduction of RGR in -Fe was generally alleviated under combined of -P-Fe conditions in *A*. *thaliana* accessions. Here, the reference accession Col-0 behaved similarly to the majority of the accessions (Fig 5B, black dot). Finally, except for few accessions including Col-0, combined -P-Zn treatment generally reversed the primary root growth reduction caused by -Zn and led to an increased RGR (Fig 5C, black dot for Col-0). This growth pattern was widespread across accessions. A few examples of the general RGR pattern are shown for accessions Shahdara, Sq-1, Sapporo-0, and Si-0 (Fig 6). Taken together, our results indicate a general mechanism in this species in which the RGR inhibition caused by single Fe and Zn deficiency is alleviated by co-occurrence with P deficiency.

### A systems biology approach identifies candidate biological processes underlying -P-Fe interactions determining root growth

**Fig 7:**
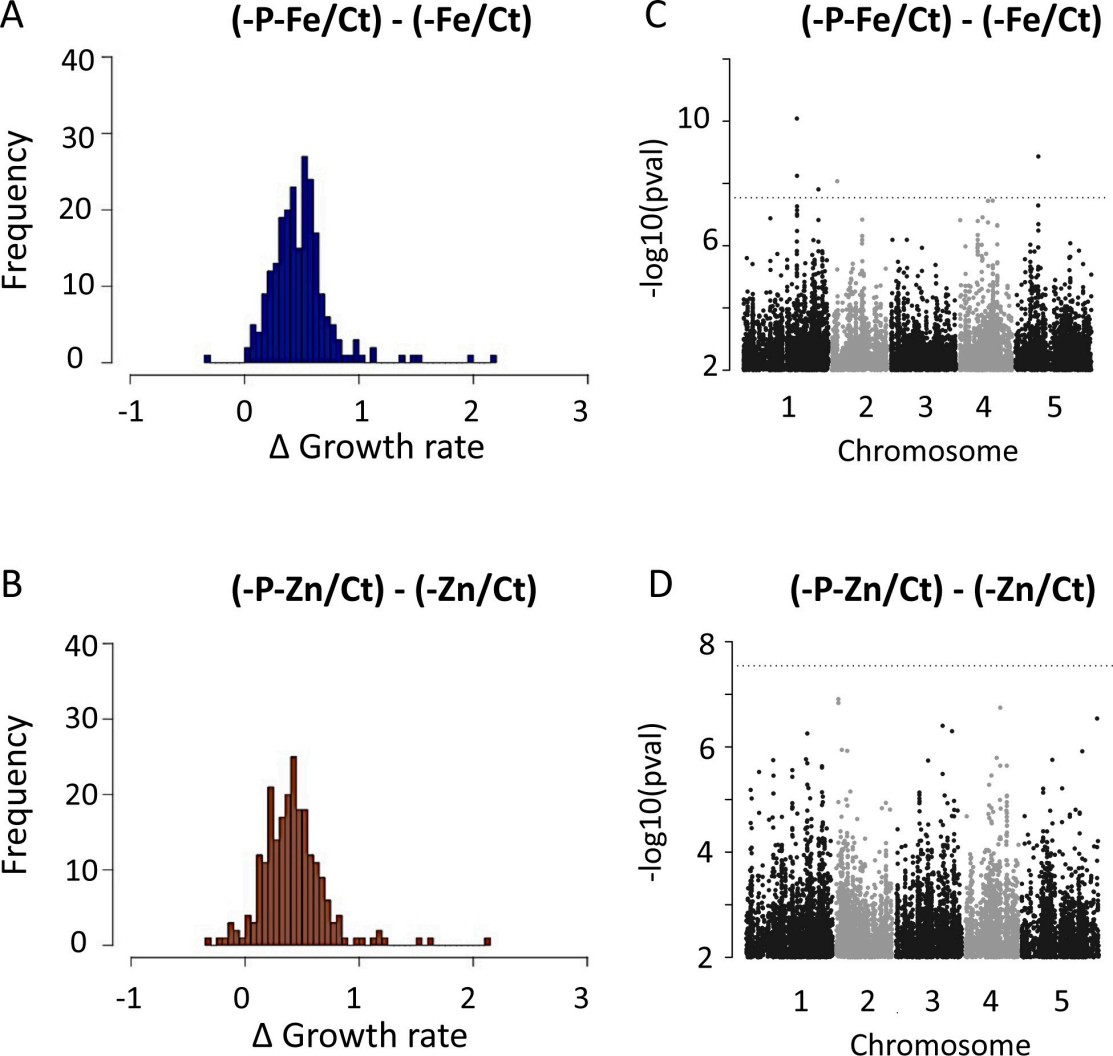
Compensation of root growth rate by nutrient limitation interactions and Manhattan plots of GWAS results. (A-B) Variation in the root growth rate (RGR) between -P-Fe and -Fe compared to control condition (Ct) presented as ΔRGR_(-P-Fe, -Fe)_ = ((-P-Fe/Ct)—RGR(-Fe/Ct)), and (B) between -P-Zn and -Zn compared to control condition (Ct)-P-Zn and–Zn presented as ΔRGR_(-P-Zn, -Zn)_ = ((-P-Zn/Ct)—RGR(-Zn/Ct)). (C) SNPs associated with the ΔRGR_(-P-Fe, -Fe)_ or (D) ΔRGR_(-P-Zn, -Zn)_. SNPs are plotted according to their position along the appropriate chromosome. Plotting colors alternate between black and grey in order to facilitate the visualization of each of the five chromosomes. 5% Bonferroni threshold is indicated by black dashed line. (C-D) Genome-wide distribution of the −log10 P-values of SNP/phenotype associations using the MMA method.

To gain insight into the genetic architecture of the mechanisms that mitigate -Fe or -Zn’s inhibition of RGR by -P, we conducted GWAS to identify loci that were associated with the variation of RGR between double and single stresses. The traits we used are as follows: ΔRGR_(-P-Fe, -Fe)_ = RGR_(-P-Fe/Ct)_—RGR_(-Fe/Ct)_, and ΔRGR_(-P-Zn, -Zn)_ = RGR_(-P-Zn/Ct)_—RGR_(-Zn/Ct)_ (Fig 7A and 7B). While the heritability for ΔRGR_(-P-Fe, -Fe)_ was estimated as 0.27, we could not determine significant heritable genetic variation for the ΔRGR_(-P-Zn, -Zn)_ trait. Consistently, using a conservative 5% Bonferroni threshold, 4 regions spanning 21 candidate genes were identified for ΔRGR_(-P-Fe, -Fe)_ (S6 Table)_,_ and no candidate genes were associated for ΔRGR_(-P-Zn, -Zn)_. From the 4 regions associated with ΔRGR_(-P-Fe, -Fe)_, 3 regions overlapped with QTLs identified in the previous analysis. The first region contains two genes, At2G05160 and AT2G05170, which were identified in the RGR for -P, -P-Fe and -P-Zn. The second and third regions span 7 and 8 genes, which were identified in the RGR for in -P-Fe and -P-Zn respectively. More importantly, the fourth region is specific to the ΔRGR_(-P-Fe, -Fe)_ trait, and contains 4 candidate genes, AT5G23950, AT5G23960, AT5G23970, and AT5G23980 that encodes a FERRIC REDUCTION OXIDASE 4, known to be involved in Fe reduction and absorption [49].

In case the above threshold was too conservative, we further screened for candidate genes and networks underlying the variation of both traits, ΔRGR_(-P-Fe, -Fe)_ and ΔRGR_(-P-Zn, -Zn)_, by using a less stringent P value threshold. We chose a −log10(P) < 4 threshold as it is frequently used in similar studies (e.g. [50,51]). Our analysis found 949 and 437 associated markers (SNP) corresponding to 186 genes and 92 genes for ΔRGR_(-P-Fe, -Fe)_ and ΔRGR_(-P-Zn, -Zn)_ respectively (S7 Table). Among the genes detected for ΔRGR_(-P-Fe, -Fe)_, we found *VITAMINC4* (*VTC4*), a gene encoding a protein with dual myo-inositol-monophosphatase and ascorbate synthase activity that has been shown to be involved in the reduction of Fe^+3^ to produce the Fe^+2^ [52]. Interestingly, VTC4 belongs to the set of genes that are direct targets of the PHOSPHARE RESPONSE1 (PHR1) [53], and is already considered as a potential candidate for the cross-talk between Fe and P signaling to regulate root growth. An overlap of 31 SNPs (S8 Table) that corresponds to 10 genes (S9 Table) was detected for ΔRGR_(-P-Fe, -Fe)_ and ΔRGR_(-P-Zn, -Zn)_. This overlap is significantly higher compared to random markers (p-value = 2.8x10^-11^) or permutations for which no significant overlap at this threshold was observed. This list includes several genes involved in the regulation of gene expression (e.g. AT1G27730, *SALT TOLERANCE ZINC FINGER*; AT1G27660, *bHLH110*) and a gene involved in DNA methylation (AT1G57820, *VARIANT IN METHYLATION 1*).

**Fig 8:**
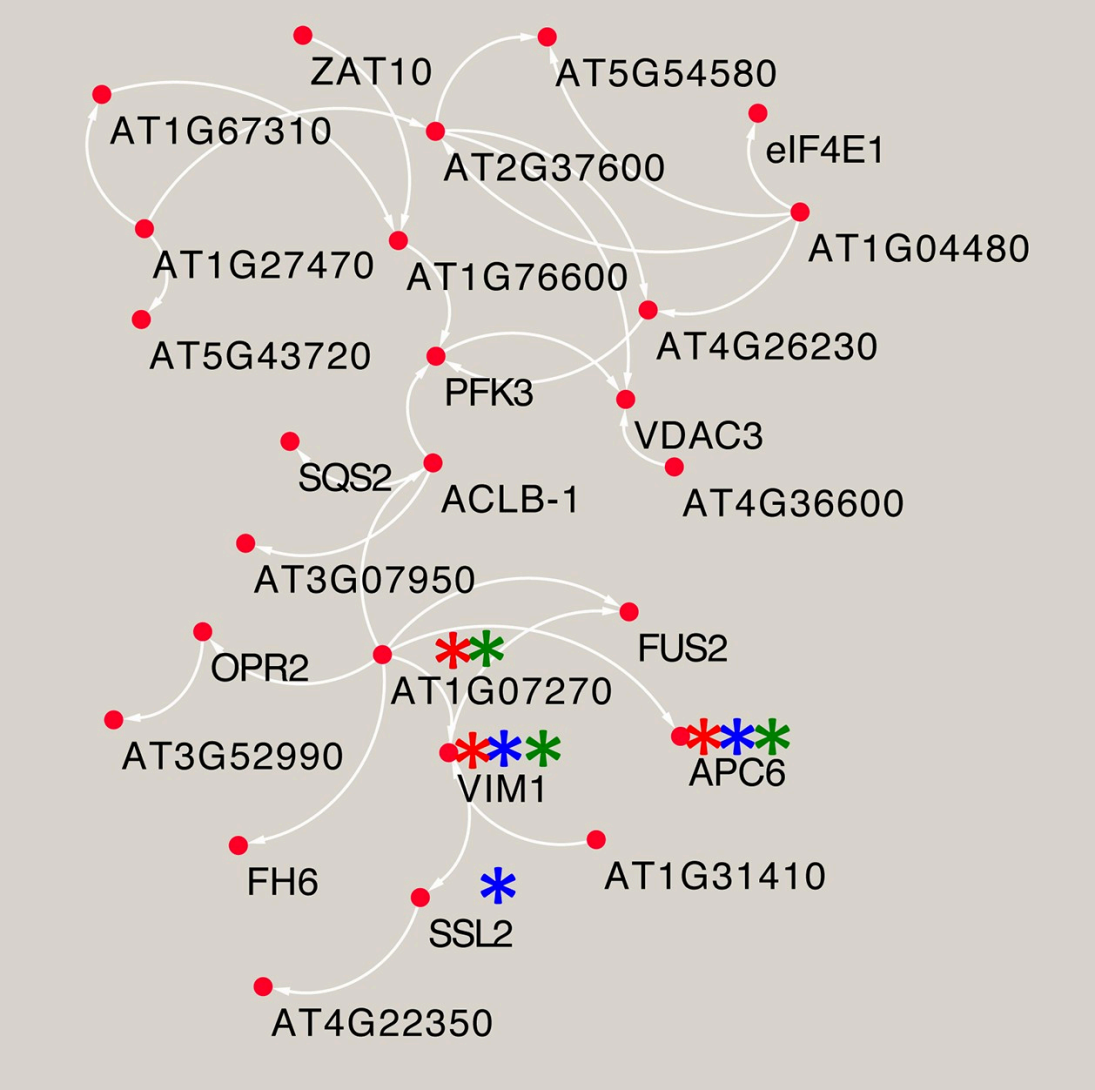
Predicted molecular pathway for the compensation of root growth rate by phosphate and iron combined stresses. A network between the subset of the GWAS candidate genes identified using the publicly available resource AraNet [56]. The network visualization is generated using Cytoscape software [79]. The Gene Ontology (GO)-biological process enrichment of a subset list of GWAS genes for ΔRGR_(-P-Fe, -Fe)_ were conducted using AraNet. Genes enriched in GO terms are marked with stars: “chromatin modification” (blue), “DNA replication” (red), and “regulation of cell cycle” (green).

To capitalize upon our GWAS results, and to go beyond our *a priori* gene list to find pathways and functional modules that explain the variation of RGR, we took advantage of the existence of the genome-scale gene co-function network AraNetv2 that covers 84% of *A*. *thaliana*’s coding genes [54]. Using this tool and all GWAS candidate genes for the ΔRGR(_-P-Fe_, _-Fe_) trait, we identified three enriched modules (S10 Table). The largest module comprises 26 genes connected with one another (Fig 8). The Gene Ontology (GO) annotation analysis revealed that there is significant enrichment of genes involved in “chromatin modification” (p-value = 2.44x10^-6^), “DNA replication” (p-value = 9.58x10^-5^), and “regulation of cell cycle” (p-value = 3.37x10^-4^) within this module. A similar analysis for genes associated with ΔRGR_(-P-Zn, -Zn)_ showed one module comprising 6 genes (S10 Table) with a significant GO enrichment for the “regulation of cell cycle” (p-value = 2.8x10^-6^) and “cell proliferation” (p-value = 3.85x10^-6^) (S10 Table). Taken together, genetic variation in genes involved in similar molecular mechanisms may be involved in promoting RGR under different combined nutrient deficiency.

### -P-Fe compensation of -Fe mediated RGR reduction involves *VARIANT IN METHYLATION 1*, *FORMIN-LIKE PROTEIN 6* and *VOLTAGE-DEPENDENT ANION-SELECTIVE CHANNEL PROTEIN 3*

**Fig 9:**
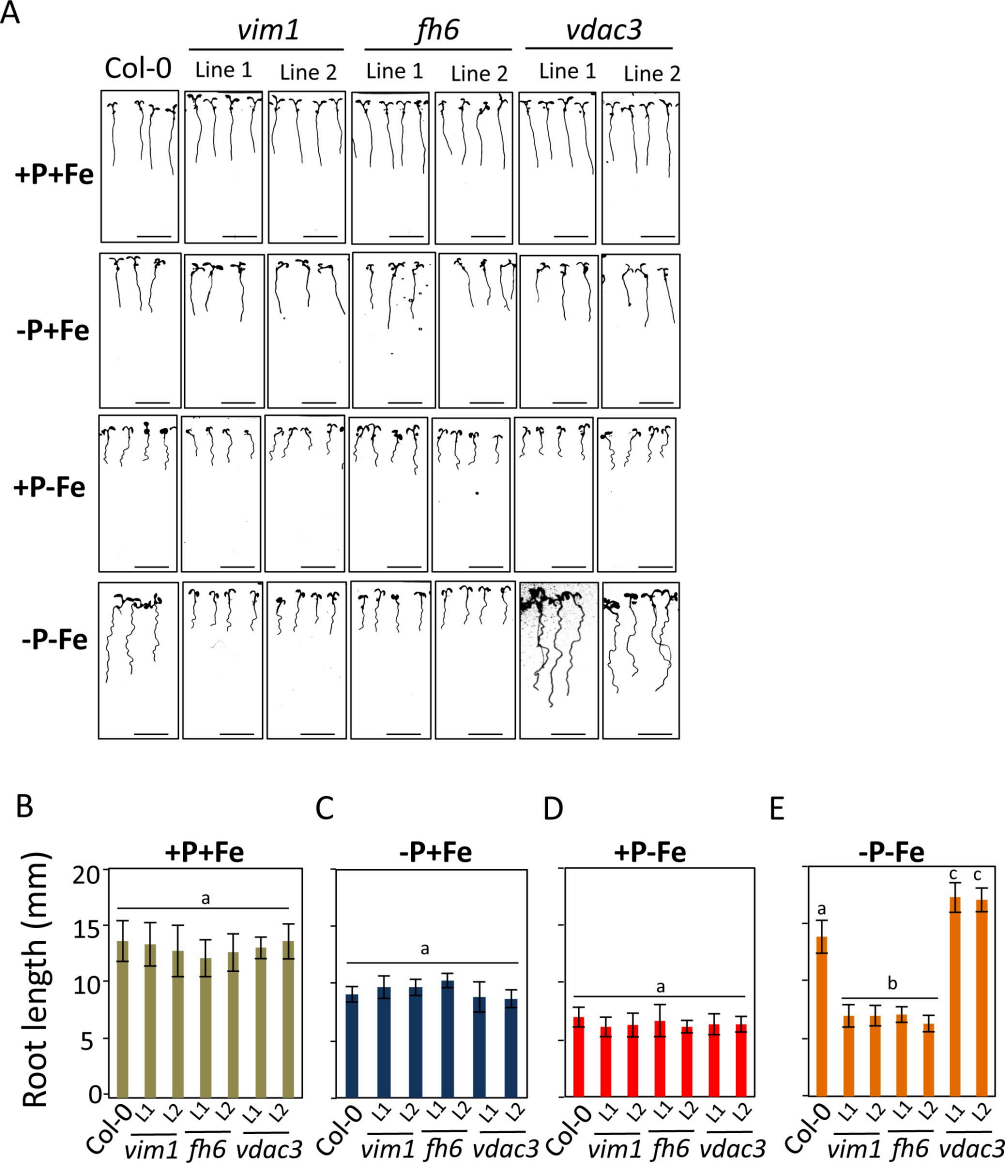
VIM1, FH6 and VDAC3 are involved in control of primary root growth under co-occurrence of low P and Fe conditions. (A) Representative primary root growth phenotypes of seedlings (day 5) grown under different growth conditions. Shown are wild-type plants (Col-0), and *vim1* (AT1G57820: line 1, SALK_050903; line 2, SALK_000930); *fh6* (AT5G67470 line1, SALK_067518; line 2, SALK_099497); *vdac3* (AT5G15090: line1, SALK_127899; line 2, N814058). Average primary root length was determined 5 days after germination on seedlings grown under control (Ct) 5B), deficiency of P (-P) (C), Fe (-Fe) (D), P and Fe (-P-Fe) (E) media. Experiments were independently repeated three times, and each data point was obtained from the analysis of primary root growth from a pool of plants (n ≥ 10). Letters indicate significantly different values at p <0.05 determined by ANOVA and Tukey HSD. L1, Line1 and L2, Line 2.

Next, we wanted to test whether our approach to combine GWAS and gene co-function network analysis could identify genes that regulate root growth under nutrient limiting conditions. For a proof of concept, we focused on the sub-network depicted in Fig 8. Twenty-five of 26 genes in this network are expressed in both roots and shoots, with the one exception of AT4G36600, which is expressed in the inflorescence (https://www.genevestigator.com/gv/). Using a reverse genetics approach, we set out to score the effect of knocking out these genes on primary root growth under different growth conditions (Ct, -Fe, -P and -P-Fe). Of the 26 genes in the network, we are able to obtain two independent T-DNA knock-out lines for 17 genes, while no knock-out line was available for 9 genes (AT1G04480: AT1G27470; AT1G76600 AT2G37600; AT3G06650; AT3G07950; AT3G52990; AT4G34650; AT5G54580). We assessed the primary root length of seedlings six days after germination. No significant difference was observed among the genotypes when grown on Ct, with the exception of mutant lines for two genes encoding for APC6 (AT1G78770) and CDC6B (AT1G07270), which are involved in cell cycle regulation and showed a severe retardation of root development. Because of these defects, we excluded these two genes from subsequent analyses. All of the remaining mutant lines showed a decrease of RGR in both -P or -Fe single treatment similar to the response of wild type plants (Col-0) (Fig 9 and S3 Fig). In Col-0 wild type plants and mutants for 11 genes, double treatment of -P-Fe lead to a restoration primary root growth to Ct-like levels. However, knock-out mutants of two genes, *VIM1* (*VARIANT IN METHYLATION 1*, AT1G57820) and *FH6* (*FORMIN-LIKE PROTEIN 6*, AT5G67470), caused a loss of this capacity. While *VIM1* had been found also in the GWAS of RGR under combined -P and -Zn stress, Col-0 and *vim1* mutants were indistinguishable in Ct, -Zn, or -P-Zn (S4 Fig), indicating its specific involvement in the response to -P-Fe combined stress. Finally, knock-out mutations of the *VDAC3* gene (*VOLTAGE-DEPENDENT ANION-SELECTIVE CHANNEL PROTEIN 3*, AT5G15090) resulted in a longer PRG under -P-Fe compared to wild type plants (Fig 9). Taken together, these genes (*VIM1*, *FH6*, and *VDAC3*) were not known to be involved in the control root growth under nutrient stress before, and their role in integrating combined -P and -Fe stress is particularly intriguing as the effect of their mutation was not visible under a single nutrient stress or control conditions.

**Fig 10:**
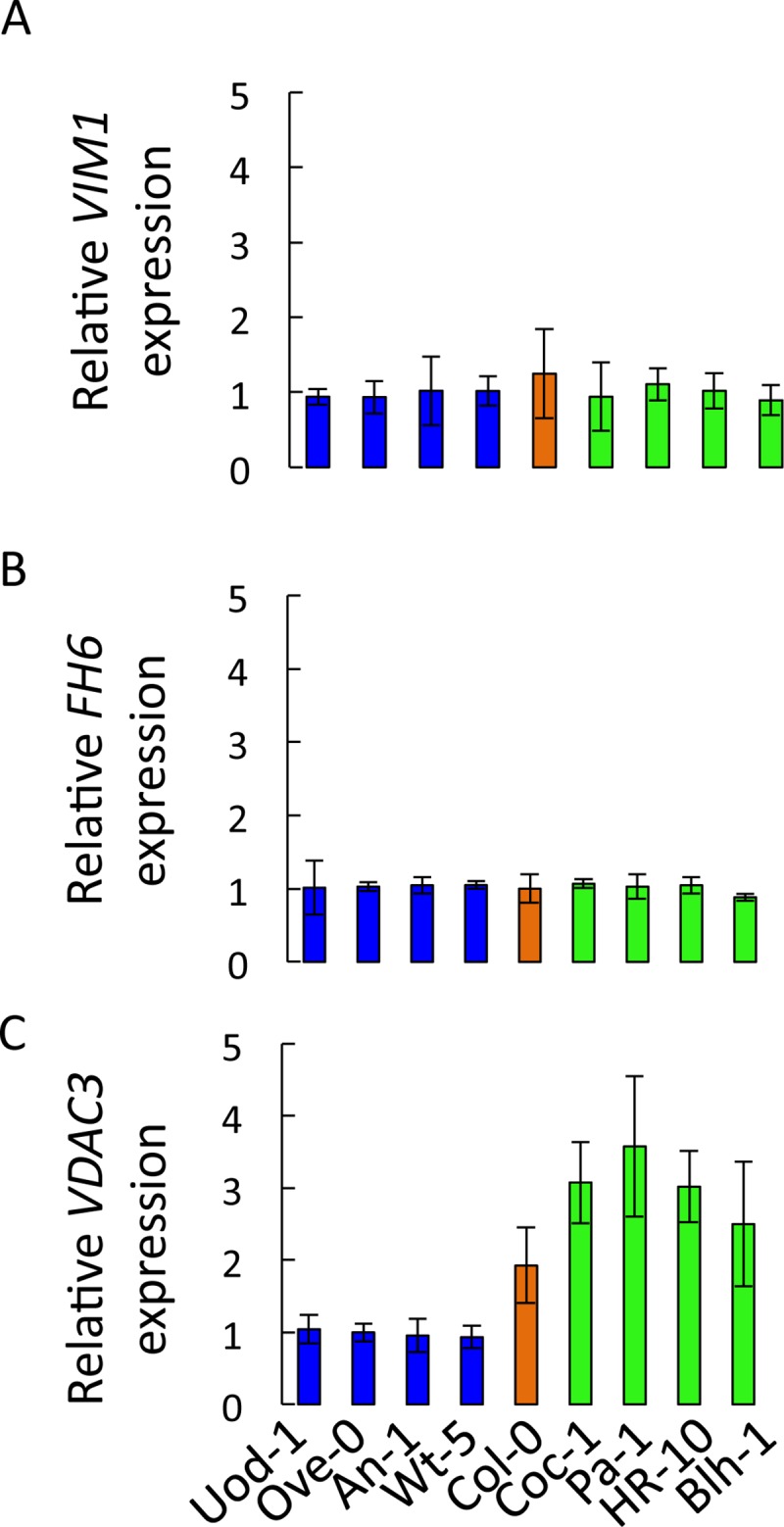
Gene expression analysis of candidate genes. Transcript accumulation of *VIM1* (A), *FH6* (B) and *VDAC3* (C) in roots of nine contrasting accessions grown for 5 days in–P-Fe conditions compared to +P-Fe conditions. Arabidopsis Ubiquitin gene was used as an internal reference. The data are given as means ± s.d. n = 10.

To obtain further insight into potential mechanisms for this involvement, we analyzed expression levels of *VIM1*, *FH6*, and *VDAC3* in Col-0 and in 8 selected accessions that had been selected according to their distinct RGR on -P-Fe conditions (high RGR in -P-Fe: Coc-1, Pa-1, HR-10, Blh-1; low RGR in -P-Fe Uod-1, Ove-0, An-1, Wt-5) and were grown under Ct or -P-Fe. Regardless of the nutritional growth condition, no difference in the accumulation of *VIM1* and *FH6* transcripts was observed in all tested genotypes (Fig 10). In contrast, *VDAC3* expression level increased significantly in accessions with high RGR in -P-Fe and remained unchanged in those displaying low RGR in -P-Fe compared to Ct condition. Further studies will be required to determine whether the differences in *VDAC3* expression are due to the natural allelic variation in the regulatory regions and/or at post-transcriptional level.

## Discussion

Below ground, roots can sense and respond to nutrient stress during all phases of plant growth and development [55]. An important part of this response is adjustment of the root growth rate in response to nutrient availability [31,56]. This plasticity of root growth is an important adaptive trait. Nevertheless, despite its prime importance for optimizing root foraging for resources in heterogeneous soil environments and ensuring crop yield, most of the work on plant mineral nutrition has focused on responses to the absence of single nutrients, and the few studies that went beyond this examined the responses only in a single accession (e.g. [31]). Here, we report the first extensive analysis of the RGR in 227 accessions of *A*. *thaliana* grown under six nutritional conditions including combinatorial nutrient deficiencies: control (MS, Ct), -P, -Fe, -Zn, -P-Fe, and -P-Zn. We focused on the variation of RGR in an early phase of plant development given its fundamental importance in the plant life cycle. We showed the presence of a large amount of heritable natural variation of RGR under each growth condition. We then performed 7 GWAS to look for the genetic variants underlying the observed natural variation of RGR. These data provide an insight into the genetic basis that underlies root growth responses to multiple nutrient deficiencies and lay a firm foundation to identify and characterize causative polymorphisms through functional molecular work.

Genetic differences between *A*. *thaliana* accessions underlie the plant’s extensive phenotypic variation, and until now these have been interpreted largely in the context of the Col-0 accession. While Col-0 has been the predominant natural accession for research in plant biology for many decades [13], our work provides clear evidence that Col-0 is not the best representative of the species for P and Zn deficiency responses. This is highly relevant because Col-0 responses were often assumed to be the general responses of plants to nutrient deficiencies. Unlike Col-0, most of the accessions reduce RGR under -Zn, and more importantly most of the accessions stimulate RGR under -P. This result indicates that Col-0 is not the ideal accession to study all types of nutrient stresses, which is in line with the exceptionality of Col-0 in root growth responses to hormonal treatments [39], and therefore broadly calls for a more comprehensive integration of other accessions in functional studies. In -P, while Col-0 consistently showed a reduction of primary root growth in many studies, the amplitude of such RGR reduction varied largely between studies. For example, in [20] Col-0 exhibited a strong response to -P (5 μM P), whereas in [41](1 μM P), the primary root is only slightly affected. On the other hand, the Shahdara accession consistently showed an oversensitivity to -P conditions [15,20,57], which was also observed in our study. This response in Shahdara was used to generate a RIL population (using Bay-0 displaying long root under -P compared to Shahdara as the other parent) that allowed the mapping of a key gene regulating primary root growth under -P, *LPR1* [20]. Despite its contrasting and consistent root growth responses to -P compared to Col-0, Shahdara remains little studied for responses to -P. In our study, we examined RGR in the early stages of plant development in response to -P in more than 200 accessions, and revealed that many accessions sense and respond to depletion of P in the medium by stimulating RGR. Since these responses were observed early on during plant development, when P levels in the seedlings will not be significantly depleted, our result suggests the existence of mechanisms that perceive changes in P and contribute to early RGR modulation by -P. These mechanisms remain to be discovered, but once discovered, they would serve as good targets to design strategies to improve root growth under -P.

Most accessions reduced RGR under -Fe or -Zn conditions. However, the extent of this reduction varied between -Fe and -Zn with -Fe causing more reduction in general. Interestingly, when each of those single deficiencies was combined with -P, we observed an increase of RGR for most of the accessions. This indicates the existence of strong interdependencies of the root growth responses between the macronutrient P and micronutrients Fe and Zn. Consistent with our results, [58] showed that the primary root length was significantly increased in response to -Fe-P compared to -Fe in Col-0. Furthermore, it has been well documented that -Fe or -Zn leads to the accumulation of P in plants [58–60], likely through the activation of root Pi uptake [60,61]. Whether the accumulation of P in -Zn or -Fe conditions is directly related to the reduction of RGR will need further investigations. Conversely, plants over-accumulate Fe in -P conditions. As aforementioned, it has been proposed that under -P, Fe over-accumulation in Col-0 roots could reach a “toxic” level that causes the inhibition of root growth. Consistently, the primary root growth grown under simultaneous absence of Fe and P is comparable to those observed under the control condition in Col-0 [18,19,40]. Our results confirm the observation made for Col-0, and extend it to a few other accessions (e.g. Shahdara, Sorbo, Rd-0, Rmx-A180, Mr-0, Hau-0, Got-22, Mdn-1, RRS-10). However, this rescue of RGR by the additional absence of -Fe was not observed in most of the accessions that we tested. Many accessions exhibited a reduction of RGR by -P-Fe compared to -P alone. This result suggests that the availability of Fe in the medium is not the sole determinant that controls RGR under -P condition. Identification of mechanism(s) that control root growth under simultaneous -P-Fe deficiency warrants deeper investigations.

Taken together, our results show that there are multiple, genetically determined strategies for how plants respond to nutrient limitations. It is therefore difficult to generalize observations made on one single accession such as Col-0 at the species level. A more comprehensive strategy is to study a variety of accessions, which is now feasible thanks to the availability of the genome sequences of thousands of accessions [62], and the possibility to generate mutations in genes of interest via gene editing technologies [63]. Our results further highlight the importance of studying combined nutrient stresses to comprehensively understand plant responses to nutrient deficiency stresses and their underlying genetic and molecular mechanisms.

Our data revealed an important aspect of plant responses to combined stresses. There is very little overlap between GWAS candidate genes from two single stresses and their combination. This result strongly suggests that there is a distinct genetic architecture underlying the responses to single and combined nutrient stresses. This is further supported by recent work exploring the natural variation in *A*. *thaliana* in response to abiotic (drought) or biotic (fungal pathogen) stresses, which indicated that distinct genetic mechanisms underlie responses to single and combined stresses [50].

Time and cost-efficiency of GWAS has made it a useful approach to understand the genetic and molecular factors that govern complex traits, such as root growth under different nutritional conditions [26,27]. Our GWAS revealed the most significant SNPs associated with the variation of RGR under each of the aforementioned six nutrient-deficient conditions. We then identified candidate genes covered by the GWAS QTLs. Some genes that are known to be involved in the regulation of Col-0 root growth under specific nutrient stress conditions were detected even with modest p-values. For example, key genes known to regulate root growth under -P or -Fe have been detected using non-conservative thresholds, namely *HPS7* (p-value = 3.83x10^-6^) for -P [17], and *bHLH104* (p-value = 2.3x10^-6^) for–Fe [25]. Interestingly, the well-characterized *LPR1* gene was identified among the GWAS candidate genes on RGR under -P (p-value = 8.18x10^-5^), -P-Fe (p-value = 2.57x10^-5^), and on ΔRGR (P-Fe, -Fe) (p-value = 3. 2x10^-5^). The *lpr1* mutant under -P is characterized by not only its longer primary root, but also its lower Fe content compared to wild-type plants [19]. Nevertheless, whether the lower Fe content in *lpr1* is enough to trigger Fe deficiency signaling is unknown. If this hypothesis were confirmed, LPR1 would play an important role in integrating both Fe and P signals to control primary root elongation. This hypothesis is supported by the recovery of root growth in Col-0 under combined -P and -Fe stresses compared to -P alone [37].

Beyond the root growth related genes, our GWAS identified many new candidate genes that were previously unknown to regulate RGR under single or combined nutrient deficiency. Nevertheless, it is important to go beyond the detection of candidate genes by GWAS either by functional validation of top ranked candidates based on p-values, or through to the identification of pathways and functional modules that help explain complex traits. In this study, starting with a list of candidate genes detected by GWAS for the compensation of RGR under -P-Fe/Ct compared to -Fe/Ct or under -P-Zn/Ct compared to -Zn/Ct [54]. Using a genome-scale gene co-function network AraNet we could predict pathways that might underlie this phenomenon (compensation of RGR). Four statistically enriched biological processes we found included the regulation of cell cycle, chromatin modification, cell proliferation, and DNA replication.

Our genetic validation of these gene modules led to the identification of three genes, *VIM1*, *FH6* and *VDAC3*, involved in restoring the Ct-like root growth rates under -P-Fe double deficiency. In line with a previous report [64], no visible growth phenotype could be observed on *vim1* null mutant plants under nutrient replete conditions. Our study showed that VIM1 is involved in regulating primary root growth under -P-Fe. The involvement of *VIM1* puts a spotlight on epigenetic processes. It has been established that epigenetic modifications, and in particular, DNA methylation, can play an important role in regulating gene expression under specific stress conditions [65]. For instance, P deficiency causes an extensive remodeling of global DNA methylation in *A*. *thaliana*, which correlates with changes in the transcript abundance of key phosphate starvation induced (PSI) genes [66]. Interestingly, the expression of several *DNA methyltransferase* genes is dependent on PHR1, a known integrator of P and Fe signals [66,67]. The requirement for *VIM1* for the appropriate responses of -P-Fe deficiency raises the possibilities that epigenetic variation may be relevant for determining for root growth in response to nutrient conditions. Moreover, Plants responds to combined nutrient stress in different manner than to a single stress [6]. This is not the simple addition of two single stresses. It has been shown that -P stress causes changes in the methylome (molecular phenotype) likely involved the *DNA methyltransferase*. Nevertheless, no visible effect was reported [66,67]. In our study, it is possible that the combination of -P and -Fe triggers a signalling pathway that regulates root growth that is not activated by the–P alone. Mutations in *FH6* also led to defects in the compensatory mechanism seen under -P-Fe for primary root growth. While mostly associated with the reorganization of the actin cytoskeleton in Arabidopsis [68–70], formin’s function is tightly intertwined with cellular signaling in animals (downstream effectors and upstream modulators of Rho GTPase signaling) [65]. It will be interesting to investigate how FH6 is molecularly involved in nutrient signal interactions. Finally, our study revealed that mutations in *VDAC3* promote root growth under -P-Fe. In Arabidopsis, VDAC3 belongs to a 4-member family called *VOLTAGE-DEPENDENT ANION-SELECTIVE CHANNEL PROTEINS—VDAC1-4*. While knock-out mutants in *VDAC1*, *VDAC2*, and *VDAC4* lead to slower plant growth, mutants in *VDCA3* are not distinguishable from wild type under standard conditions [71], which is in agreement with our results. Overexpression of *VDAC*3 leads to a very short primary root under abiotic stress (e.g. NaCl treatment) [72]. It has been proposed that the closure of the VDAC3 channel reduces the generation of reactive oxygen species (ROS) [72]. Interestingly, ROS was proposed to be main cause of root growth inhibition under–P [73]. A possible hypothesis is therefore that loss of *VDAC3* leads to a lower amount of ROS, which in turn could contribute to explain the longer root under the combined stress -P-Fe. Taken together, our systems genetics approach showed that VIM1, FH6 and VDAC3 are involved in regulating root growth under the -P-Fe deficiency. Nevertheless, it is important to mention that although mutations of other predicted genes in Col-0 background have not shown detectable phenotypes under -P, -Fe or -P-Fe, this does not rule out the possibility that these genes might have roles on regulating the root growth in other *A*. *thaliana* accessions. In particular, we would predict that due to the outlier position of Col-0, many functionally relevant genes have been overlooked in forward and reverse genetic studies in Col-0. Therefore, for future studies great promise lies in studying different accessions and in particular to characterize gene function of genetic variants in these. This is now possible as the still rapidly developing CRISPR/CaS9 technology not only allows for generating mutants in different accessions but also for editing variants or even replacing genes directly in accessions with interesting alleles [74]. The outlier position of Col-0 in growth responses to nutrient deficiencies (Fig 3) and to hormone responses [39], as well as the abundance and relevance of natural loss of function mutations [75], highlights the need of using genome-scale networks, such as Aranet, on an expanded data basis, which should include data from diverse accessions. This promises to unleash the full power of systems genetics to potentially accelerate future research discovery leading to the improvement of our understanding of the regulation of plant growth under unfavorable conditions.

## Materials and methods

### Plant materials and growth conditions

A total of 227 natural accessions of *A*. *thaliana* (S1 Table, columns 1 and 2) were phenotyped and used to perform GWAS. SALK T-DNA insertion mutant lines used in the study were obtained from Nottingham Arabidopsis Stock Centre [64]: AT1G07270 (SALK_128156); AT1G27730 (SALK_054092); AT1G31410 (SALK_013525); AT1G57820 (SALK_050903, SALK_000930); AT1G67310 (SALK_087870); AT1G76690 (SALK_014855); AT1G78770 (SALK_008789); AT2G25170 (SALK_033554); AT4G18040 (SALK_145583); AT4G22350 (SALK_132163); AT4G26230 (SALK_040183); AT4G26270 (SALK_095751); AT4G36600 (SALK_046270); AT5G15090 (SALK_127899, N814058), AT5G43720 (SALK_000441); AT5G67470 (SALK_067518, SALK_099497). We used PCR-based screening to genotype and confirm the absence of transcripts of each mutated gene. The PCR primers used in this study are listed in S11 Table. Six nutritional conditions were used, MS (Control; Ct), phosphate deficiency (-Pi), iron deficiency (-Fe), zinc deficiency (-Zn), phosphate and iron deficiency (-Pi-Fe) and phosphate and zinc deficiency (-Pi-Zn). Seeds were surface-sterilized in chlorine gas for 1 h. Chlorine gas was generated from 130 ml of 10% sodium hypochlorite and 3.5 ml of 37% hydrochloric acid. After sterilization, seeds were imbibed in water and stratified for 3 days at 4 °C in the dark to promote uniform germination. For each genotype we sowed 12 seeds, distributed on 4 different plates. Each plate contained eight different accessions with 3 plants per accession. Plates were placed in the growth chamber in a randomized manner. Seeds were then germinated and grown vertically on 1X MS-agar medium, which contained 1 mM KH_2_PO_4_, 1 mM MgSO_4_, 0.5 mM KNO_3_, 0.25 mM Ca(NO_3_)_2_, 10 μM MnCl_2_, 30 μM H_3_BO_3_, 1 μM CuCl_2_, 0.1 μM (NH_4_)_6_Mo_7_O_24_, 50μM KCl, 100 μM NaFeEDTA and 15 μM ZnSO_4_ in presence of 0.8% (wt/vol) agar and 1% (wt/vol) sucrose. -Pi medium was made by replacing the source of Pi (KH_2_PO_4_) to with 1 mM KH_2_CaCO_3_. -Zn medium was made by omitting the source of Zn (ZnSO_4_) in the medium. -P-Zn medium was made by not adding the only source of Zn (ZnSO_4_) and by replacing KH_2_PO_4_ with 1 mM KCl to the medium. -Fe medium was made by not adding the FeEDTA in the medium and by supplying FerroZine that is known as a strong Fe chelator. -P-Fe medium was made by not adding the source of Fe (FeEDTA) and by supplying FerroZine, and by replacing the P source (KH_2_PO_4_) by 1mM KH_2_CaCO_3_ to the medium. Plants were grown at 22°C in the same growth chambers under the same light regime (long-day: 8 h dark, 16 h light) conditions. Plant phenotyping for GWAS was performed as described previously [40]. The BRAT software was used to perform root trait quantification [40].

### Genome wide association studies (GWAS)

Genome-wide association mapping was performed on the regression coefficients of root growth of three-, four- and five-days-old seedlings grown under the above detailed conditions. The phenotypic data for the PRL of the seedlings as well as the estimated root growth rate (RGR) are shown in S1 Table. The normalized RGR used in the analysis are shown in S2 Table. All phenotypic data are also available at the AraPheno database [76]. The genotypic data were based on whole genome sequencing data [The 1001 Genomes Consortium, 2016] and covered 4,932,457 SNPs for the 227 accessions. 1,739,142 of these markers had a minor Allele frequency of at least 5% in the population and where further used for GWAS. GWAS was performed with a mixed model correcting for population structure (emmaX, [77]) The kinship structure has been calculated under the assumption of the infinitesimal model using all genetic markers with a minor Allele Frequency of more than 5% in the whole population. The analysis was performed in R (R Core Team (2016)). The used R scripts are available at https://github.com/arthurkorte/GWAS. The Genotype Data used for GWAS are available www.1001genomes.org. All GWAS results are also available in the AraGWAS catalog [76].

Heritability estimates have been extracted from the mixed model according to the formula: H^2^ = V_G_ /(V_G_ + V_E_), where V_G_ is the among-genotype variance component and V_E_ is the residual (error) variance.

### Molecular pathway prediction

The functional modules were predicted based on the GWAS genes using the publicly available resource AraNetv2 [54]. Network visualization was generated using Cytoscape software (version 3.3.0) [78].

### Gene expression analysis by real-time quantitative reverse-transcription PCR

Arabidopsis wild type plants of nine accessions were grown in presence or absence of P and Fe for 5 days. Two μg of total RNA extracted from roots were used to synthesize cDNA using poly-A oligos. RNA extraction, reverse transcription, and real-time quantitative reverse-transcription PCR was performed as described [45]. The primer list is provided in S11 Table. For every sample, the relative gene expression of each gene was expressed following normalization against the cycle threshold values obtained for the gene used for standardization, and the fold change in relative gene expression. PCR reactions were performed in triplicates. For each gene, the relative amount of calculated mRNA was normalized to the level of the control gene *ubiquitin10* mRNA (*UBQ10*: At4g05320).

## Supporting information

S1 Table Primary root length (in pixels) and root growth rate (RGR) of 227 natural accessions of *Arabidopsis thaliana* grown on control condition (Ct), deficiency of phosphorus (-P), iron (-Fe), zinc (-Zn), phosphorus and iron (-P-Fe), or phosphorus and zinc (-P-Zn). Measurements were done on 3, 4 and 5 days-old seedlings. The presented values are the mean of twelve replicates per accession and treatment.
(TIF)

S2 Table Normalized root growth rate (RGR) of the 227 natural *Arabidopsis thaliana* accessions. Values were obtained by dividing the RGR presented in S1 Table for each of the nutrient-deficient conditions with the RGR under control condition (RGRnorm(X/Ct), column 2–6) or for the combination of -P -Fe additionally dividing the RGR against the RGR under -Fe (RGRnorm(PFe/Fe), column 7) and for the combination of -P -Zn against the RGR under -Z (RGRnorm(PZn/Zn), column 8).
(TIF)

S3 Table Heritability estimates for the root growth rate (RGR) and the normalized RGR using estimates from the linear mixed model. (TIF)

S4 Table List of 145 candidate genes, corresponding to 32 different genomic regions that are present in a 10kb window around significant SNPs for the GWAS of normalized root growth rate (RGR) under nutrient-deficient conditions. The threshold used to declare these SNPs significant is 5% Bonferroni. If the gene was found in the respective analysis, it is denoted with a 1 in the table, where a value of 0 indicates no significant association.
(TIF)

S5 Table List of 87 significant SNPs for the GWAS of normalized root growth rate (RGR) under the different nutrient-deficient conditions. The table shows the position of the respective SNPs, the minor Allele frequency (MAF), a binary value (0/1) indicating whether the SNP was significant in the respective analysis, where a value of 1 denotes a significant association and a value of 0 indicates no association, and the genes that are at this position or in a 10kb window surrounding these SNPs, respectively.
(XLS)

S6 Table List of 21 candidate genes found in the analyses of root growth rate (RGR) of phosphorus and iron deficiency (-P-Fe) normalized on iron deficiency (-Fe). (XLSX)

S7 Table List of candidate genes found in the GWAS analyses (threshold of p = 10^−4^) of root growth rate (RGR) of phosphorus and iron deficiency (-P-Fe) normalized on iron deficiency (-Fe) and phosphorus and zinc deficiency (-P-Zn) normalized on zinc deficiency (-Zn). (XLS)

S8 Table List of 31 SNPs that are found in the GWAS analyses (threshold of p = 10^−4^) of RGR of phosphorus and iron deficiency (-P-Fe) normalized on iron deficiency (-Fe) and phosphorus and zinc deficiency (-P-Zn) normalized on zinc deficiency (-Zn). (XLS)

S9 Table List of 10 genes that are found in both, the analyses of RGR of phosphorus and iron deficiency (-P-Fe) normalized on iron deficiency (-Fe) and phosphorus and zinc deficiency (-P-Zn) normalized on zinc deficiency (-Zn) at a less stringent threshold of p = 10^−4^. (XLS)

S10 Table List of genes forming the modules identified through the analyses of GWAS candidate genes for ΔRGR_(-P-Fe, -Fe)_ and for ΔRGR_(-P-Zn, -Zn)_ traits using the publicly available resources, AraNet. (XLSX)

S11 Table List of primers used in this study. (XLSX)

S1 Fig Effect of single and double deficiencies of iron, zinc and phosphorus on the primary root elongation of the *Arabidopsis thaliana* reference accession Col-0. Seeds of the *A*. *thaliana* Col-0 accession were germinated on six different nutrient conditions: control (Ct), deficiency of P (-P), Fe (-Fe), Zn (-Zn), P and Fe (-P-Fe), and P and Zn (-P-Zn). The primary root length was determined on 3-, 4-, and 5-day-old seedlings.
(TIF)

S2 Fig Comparison of GWAS for normalized- and non-normalized RGR under–P. SNP association P-values of GWAS for normalized RGR under -P are plotted against these of the non-normalized RGR under -P. Each dot represents one of the 1.7M SNPs tested in the analysis, where -log10 of the respective p-value is plotted. Only SNPs with a p-value < 0.01 in at least one of the two analyses are included in the plot. The dotted red line represents the 5% Bonferroni threshold that was used to declare markers as significant.
(TIF)

S3 Fig Primary root growth of seedlings grown in the presence or absence of P and/or Fe. Arabidopsis Col-0 and eleven T-DNA mutant lines were germinated in four different nutrient conditions: control (Ct) (A), deficiency of P (-P) (B), Fe (-Fe) (C), P and Fe (-P-Fe) (D). Average primary root length of each genotype was determined 5 days after germination. Mutations were in the following genes: AT1G27730 (SALK_054092); AT1G31410 (SALK_013525); AT1G67310 (SALK_087870); AT1G76690 (SALK_014855); AT2G25170 (SALK_033554); AT4G18040 (SALK_145583); AT4G22350 (SALK_132163); AT4G26230 (SALK_040183); AT4G26270 (SALK_095751); AT4G36600 (SALK_046270); AT5G43720 (SALK_000441). Experiments were independently repeated three times, and each data point was obtained from the analysis of primary root growth from a pool of plants (n ≥ 10).
(TIF)

S4 Fig Primary root growth of seedlings grown in the presence or absence of P and/or Zn. Arabidopsis Col-0 and *vim1* mutant lines were germinated three different nutrient conditions: control (+P+Zn) (A), -Zn (B) and -P-Zn (C). Average primary root length of each genotype was determined 5 days after germination. Experiments were independently repeated three times, and each data point was obtained from the analysis of primary root growth from a pool of plants (n ≥ 10).
(TIF)
