# Correction: Systems genomics approaches provide new insights into *Arabidopsis thaliana* root growth regulation under combinatorial mineral nutrient limitation

**DOI:** 10.1371/journal.pgen.1009107

**Published:** 2020-09-23

**Authors:** The PLOS Genetics Staff

S1–S4 Tables are omitted from the list of Supplementary Information. S1–S4 Tables incorrectly link to S1-4 Figs. Please view the correct S1–S4 Tables below. The publisher apologizes for the error.

## Supporting information

S1 Table Primary root length (in pixels) and root growth rate (RGR) of 227 natural accessions of Arabidopsis thaliana grown on control condition (Ct), deficiency of phosphorus (-P), iron (-Fe), zinc (-Zn), phosphorus and iron (-P-Fe), or phosphorus and zinc (-P-Zn). Measurements were done on 3, 4 and 5 days-old seedlings. The presented values are the mean of twelve replicates per accession and treatment.
(XLS)

S2 Table Normalized root growth rate (RGR) of the 227 natural *Arabidopsis thaliana* accessions. Values were obtained by dividing the RGR presented in S1 Table for each of the nutrient-deficient conditions with the RGR under control condition (RGRnorm(X/Ct), column 2–6) or for the combination of -P -Fe additionally dividing the RGR against the RGR under -Fe (RGRnorm(PFe/Fe), column 7) and for the combination of -P -Zn against the RGR under -Z (RGRnorm(PZn/Zn), column 8).
(XLS)

S3 Table Heritability estimates for the root growth rate (RGR) and the normalized RGR using estimates from the linear mixed model. (XLSX)

S4 Table List of 145 candidate genes, corresponding to 32 different genomic regions that are present in a 10kb window around significant SNPs for the GWAS of normalized root growth rate (RGR) under nutrient-deficient conditions. The threshold used to declare these SNPs significant is 5% Bonferroni. If the gene was found in the respective analysis, it is denoted with a 1 in the table, where a value of 0 indicates no significant association.
(XLSX)
