# Supplementary figures and images for: Systems genomics approaches provide new insights into Arabidopsis thaliana root growth regulation under combinatorial mineral nutrient limitation
Source: PLoS Genet. 2019 Nov 6;15(11):e1008392. doi: 10.1371/journal.pgen.1008392 (PMC6834251; doi:10.1371/journal.pgen.1008392)

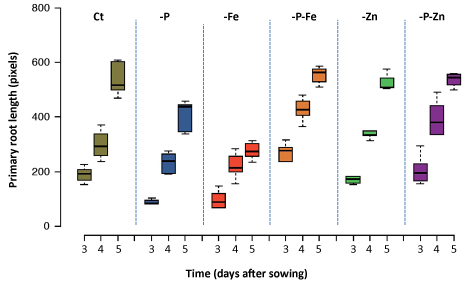

Supplement: S1 Table — The presented values are the mean of twelve replicates per accession and treatment. (TIF) [file pgen.1008392.s001.tif]

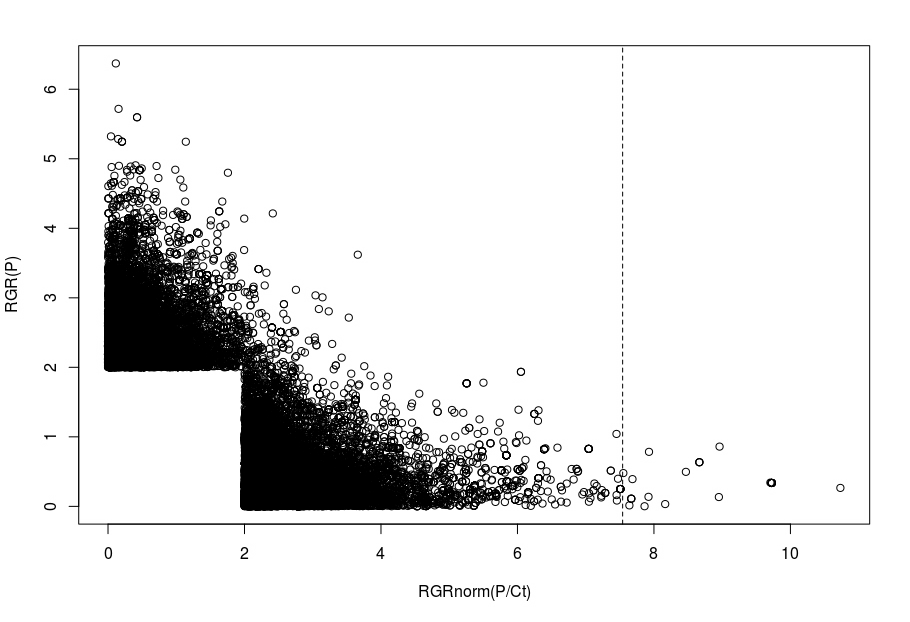

Supplement: S2 Table — Values were obtained by dividing the RGR presented in S1 Table for each of the nutrient-deficient conditions with the RGR under control condition (RGRnorm(X/Ct), column 2–6) or for the combination of -P -Fe additionally dividing the RGR against the RGR under -Fe (RGRnorm(PFe/Fe), column 7) and for the combination of -P -Zn against the RGR under -Z (RGRnorm(PZn/Zn), column 8). (TIF) [file pgen.1008392.s002.tif]

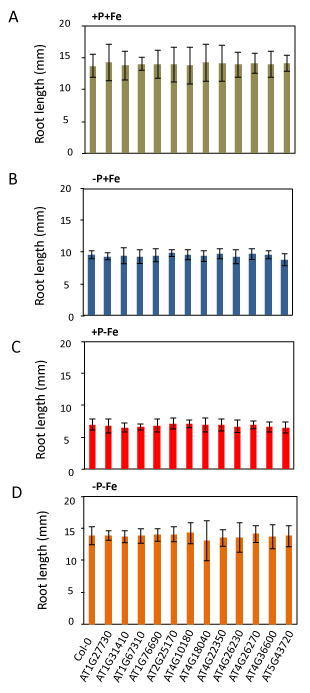

Supplement: S3 Table — (TIF) [file pgen.1008392.s003.tif]

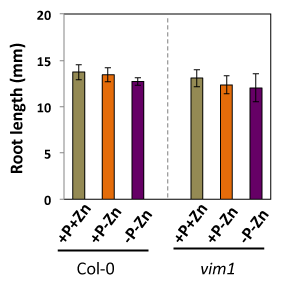

Supplement: S4 Table — The threshold used to declare these SNPs significant is 5% Bonferroni. If the gene was found in the respective analysis, it is denoted with a 1 in the table, where a value of 0 indicates no significant association. (TIF) [file pgen.1008392.s004.tif]

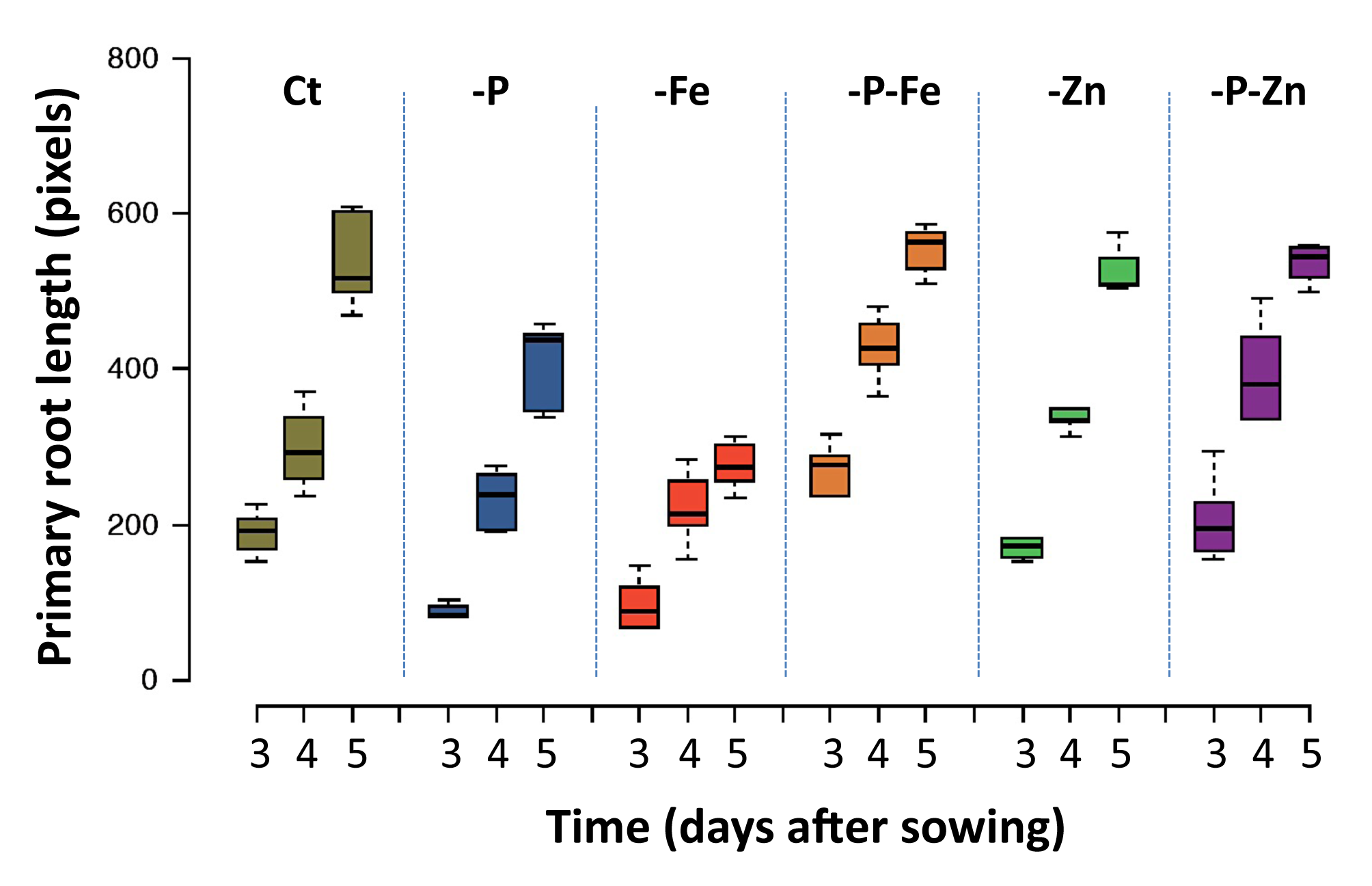

Supplement: S1 Fig — Seeds of the A. thaliana Col-0 accession were germinated on six different nutrient conditions: control (Ct), deficiency of P (-P), Fe (-Fe), Zn (-Zn), P and Fe (-P-Fe), and P and Zn (-P-Zn). The primary root length was determined on 3-, 4-, and 5-day-old seedlings. (TIF) [file pgen.1008392.s012.tif]

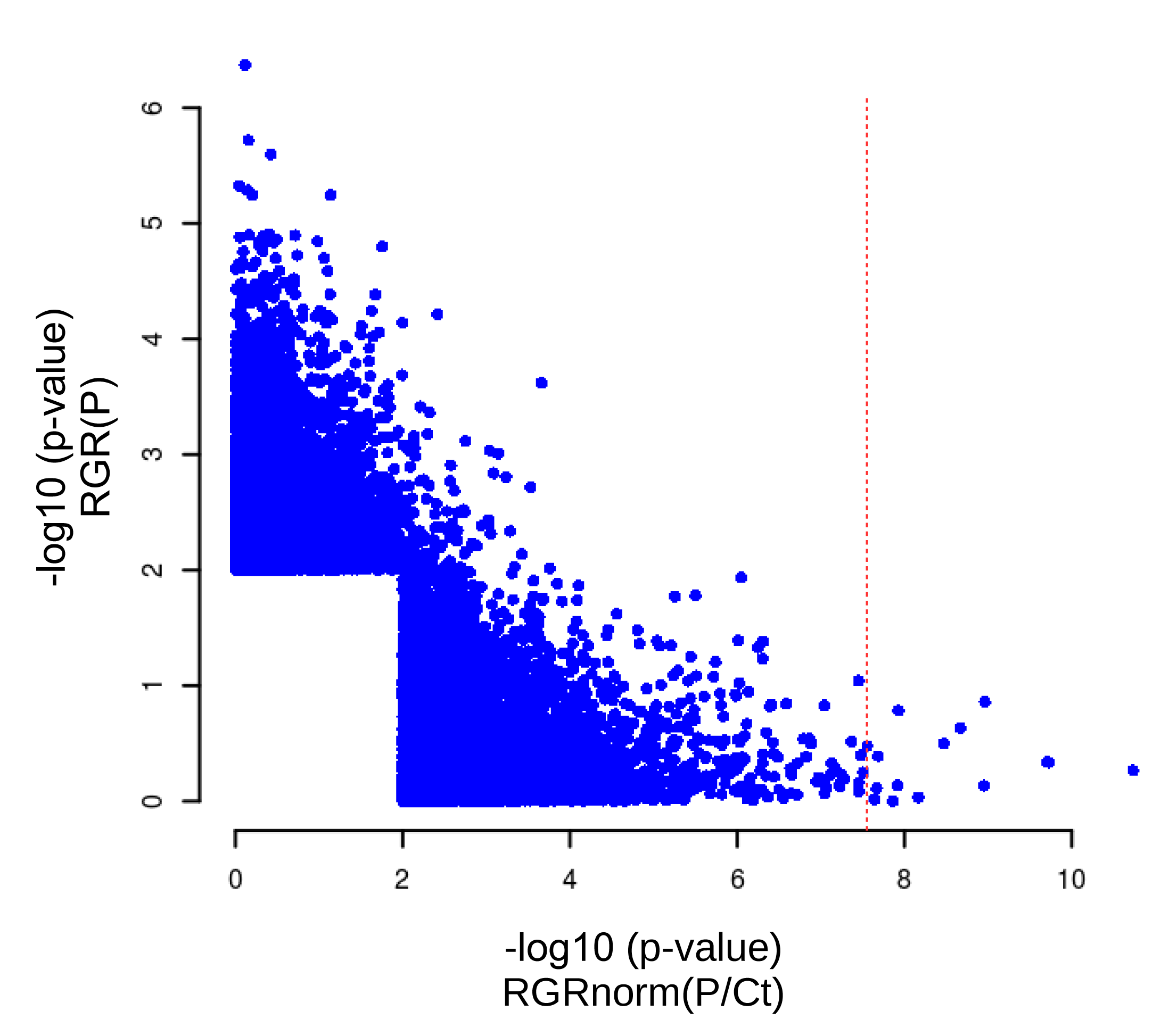

Supplement: S2 Fig — SNP association P-values of GWAS for normalized RGR under -P are plotted against these of the non-normalized RGR under -P. Each dot represents one of the 1.7M SNPs tested in the analysis, where -log10 of the respective p-value is plotted. Only SNPs with a p-value < 0.01 in at least one of the two analyses are included in the plot. The dotted red line represents the 5% Bonferroni threshold that was used to declare markers as significant. (TIF) [file pgen.1008392.s013.tif]

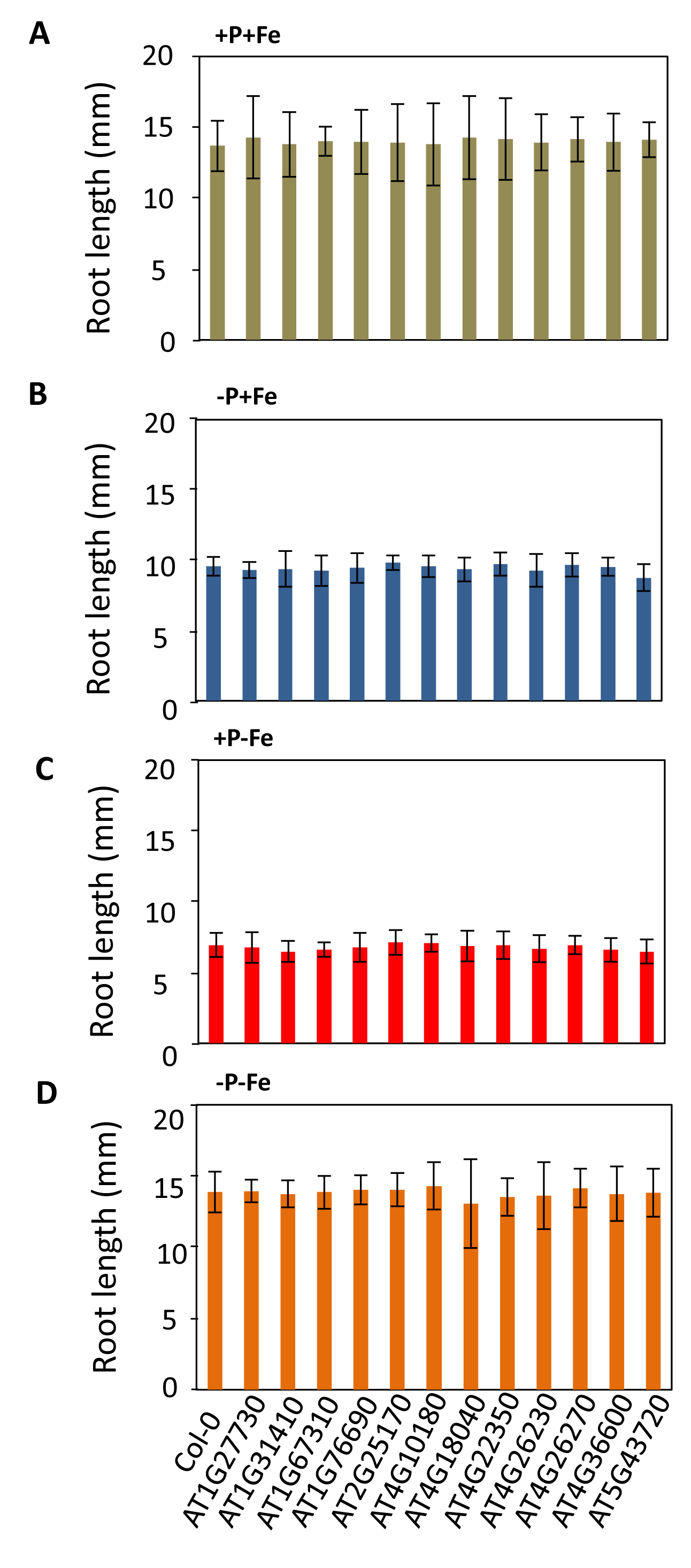

Supplement: S3 Fig — Arabidopsis Col-0 and eleven T-DNA mutant lines were germinated in four different nutrient conditions: control (Ct) (A), deficiency of P (-P) (B), Fe (-Fe) (C), P and Fe (-P-Fe) (D). Average primary root length of each genotype was determined 5 days after germination. Mutations were in the following genes: AT1G27730 (SALK_054092); AT1G31410 (SALK_013525); AT1G67310 (SALK_087870); AT1G76690 (SALK_014855); AT2G25170 (SALK_033554); AT4G18040 (SALK_145583); AT4G22350 (SALK_132163); AT4G26230 (SALK_040183); AT4G26270 (SALK_095751); AT4G36600 (SALK_046270); AT5G43720 (SALK_000441). Experiments were independently repeated three times, and each data point was obtained from the analysis of primary root growth from a pool of plants (n ≥ 10). (TIF) [file pgen.1008392.s014.tif]

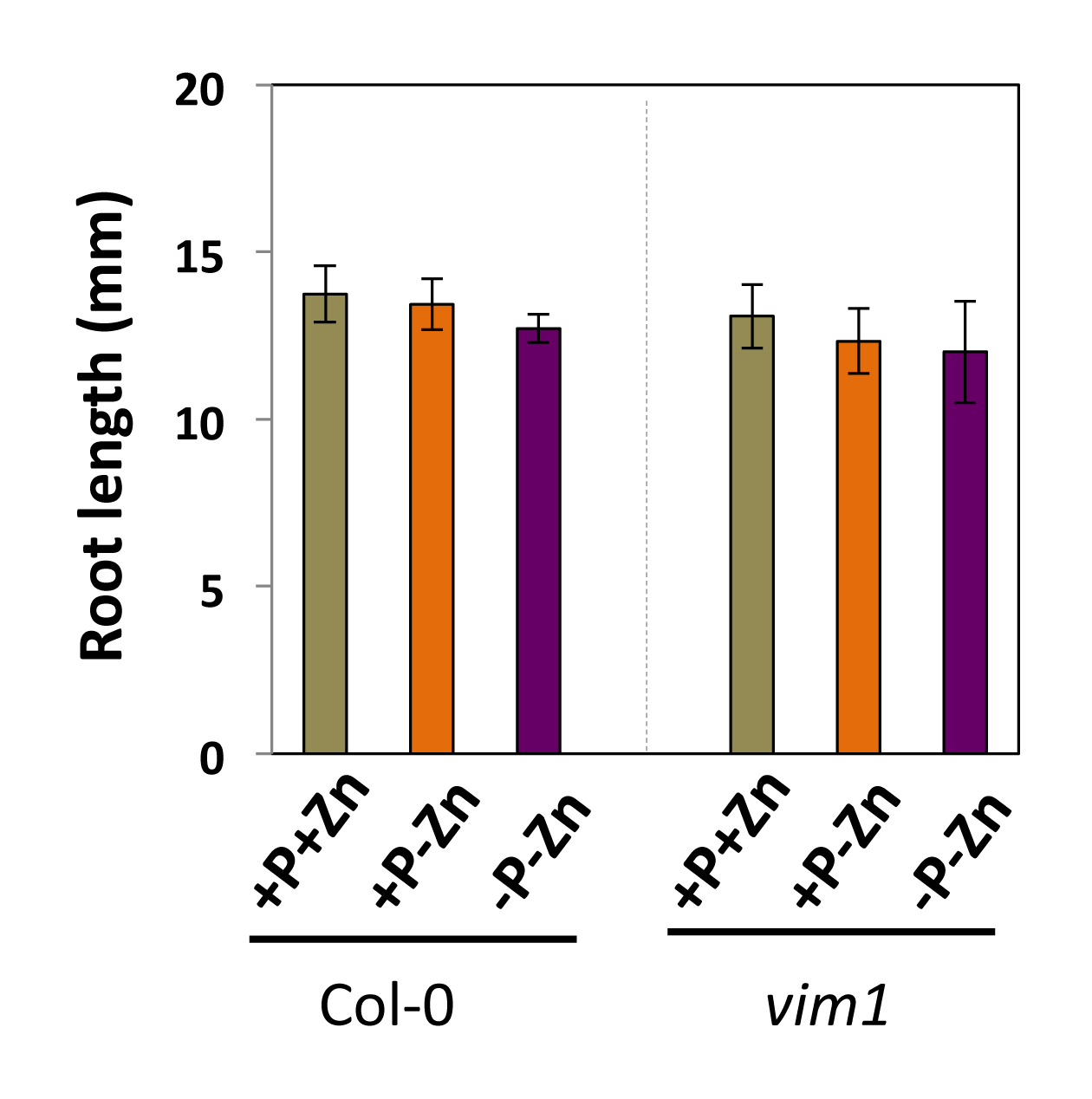

Supplement: S4 Fig — Arabidopsis Col-0 and vim1 mutant lines were germinated three different nutrient conditions: control (+P+Zn) (A), -Zn (B) and -P-Zn (C). Average primary root length of each genotype was determined 5 days after germination. Experiments were independently repeated three times, and each data point was obtained from the analysis of primary root growth from a pool of plants (n ≥ 10). (TIF) [file pgen.1008392.s015.tif]
